# Molecular architecture with carbohydrate functionalized β-peptides adopting 3_14_-helical conformation

**DOI:** 10.3762/bjoc.10.93

**Published:** 2014-04-28

**Authors:** Nitin J Pawar, Navdeep S Sidhu, George M Sheldrick, Dilip D Dhavale, Ulf Diederichsen

**Affiliations:** 1 Institute for Organic and Biomolecular Chemistry, Georg-August University Göttingen, Tammannstrasse 2, D-37077 Göttingen, Germany; 2 Department of Chemistry, Garware Research Centre, University of Pune, Pune 411 007, India; 3 Institute for Inorganic Chemistry, Georg-August University Göttingen, Tammannstraße 4, D-37077 Göttingen, Germany

**Keywords:** carbohydrate recognition, conformation, glycopeptide, β-peptide, sugar amino acid

## Abstract

Carbohydrate recognition is essential in cellular interactions and biological processes. It is characterized by structural diversity, multivalency and cooperative effects. To evaluate carbohydrate interaction and recognition, the structurally defined attachment of sugar units to a rigid template is highly desired. β-Peptide helices offer conformationally stable templates for the linear presentation of sugar units in defined distances. The synthesis and β-peptide incorporation of sugar-β-amino acids are described providing the saccharide units as amino acid side chain. The respective sugar-β-amino acids are accessible by Michael addition of ammonia to sugar units derivatized as α,β-unsaturated esters. Three sugar units were incorporated in β-peptide oligomers varying the sugar (glucose, galactose, xylose) and sugar protecting groups. The influence of sugar units and the configuration of sugar-β-amino acids on β-peptide secondary structure were investigated by CD spectroscopy.

## Introduction

Synthetic biomimetic macromolecules, which are capable to fold into well-defined three-dimensional structures in analogy to natural peptides and proteins, have been extensively studied to increase the understanding of complex biomolecules [1–3]. In this respect, β-peptides are especially of interest as peptidomimetic foldamers wherein the presence of β-amino acids rendered them to adopt a variety of conformational stable secondary structures, even with short peptide sequences [4–6]. Amongst them the 3_14_-helix is the most significant helical secondary structure in β-peptides requiring three amino acids per turn and orienting every third side chain (*i* and *i*+3) on the same side of the helix [7–11]. β-Peptide helices are stable in water or organic solvents and are highly resistant towards enzymatic degradation [12]. Furthermore, they provide sheet-like structures and can be used for helical self-association towards protein-like assemblies mimicking secondary structures and eventually acting as inhibitors for protein–protein interaction [13–14]. In addition, β-peptide 3_14_-helices furnish an ideal structural backbone for the well-organized presentation of recognition units since incorporation of artificial β-amino acids allows positioning of side chains on one side of the helix in equidistant 5 Å intervals. This concept was proven to be beneficial for base-pair recognition of β-peptide nucleic acids leading to high duplex stabilities of entropically preorganized recognition units [15–17]. Further, the presentation of a sugar unit on a β-peptide helical topology was reported by Arvidsson and coworkers [18–19]. One D-galactose unit is positioned on the helical surface taking advantage of peptide folding for biomolecular interaction with corresponding lectins. Taillefumier and coworkers link sugar units to β-peptide amino acid side chains by azide–alkyne cycloaddition [20].

**Figure 1:**
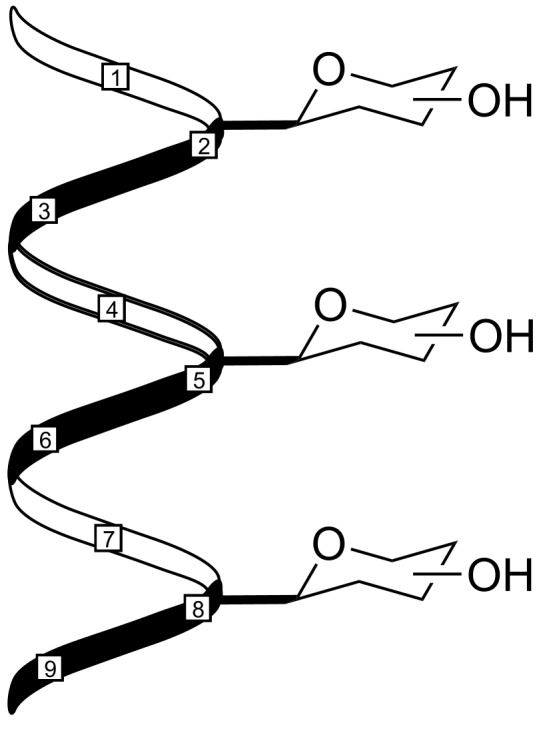
Sketch of right-handed β-peptide helix functionalized in every third amino acid by carbohydrates presenting equidistant sugar units with uniform orientation in 5 Å intervals.

Following the concept of highly organized presentation of sugar units on a β-peptide scaffold, we report on simultaneous incorporation of various sugars (glucose, galactose, xylose) as sugar-β-amino acids in a 3_14_-helix. Up to three sugar units were linearly aligned with 5 Å distance (Figure 1). This kind of sugar organization will be of later relevance, e.g., in lectin binding studies and with respect to the investigation of multivalency effects [21–23].

The use of peptide scaffolds for the presentation of sugar epitopes has already some precedence. Complex saccharide structures are linked to α-peptides, protein fragments [24–27] and recently also to glycofoldamers [28]. Sugars are arranged on cyclopeptides [29–33], like Dumy and coworkers report well-defined tetravalent mannose glycoconjugates on a cyclic peptide which show specific binding with concanavalin A [29]. Further, the ternary type-II polyproline helix is used for the structurally defined presentation of sugar units [34], and similarly the β-peptides provide a suitable conformationally constrained and well-defined scaffold for sugar presentation on a 3_14_-helix [18–20] as well as on a β-peptide 3_12_-helical scaffold obtained by oligomerization of glycosylated pyrrolidine β-amino acids [35].

Glycopeptide or glycoprotein synthesis is challenged by different conditions required for carbohydrate and peptide chemistry. Therefore, sugar units are introduced by side-chain ligation and labeling strategies on the peptide scaffold or were established by incorporation of sugar-β-amino acids by solid-phase peptide synthesis (SPPS) [36–37]. Sugar amino acid building blocks have attracted interest due to their use as structural elements as peptidomimetics [38–39], oligosaccharide mimetics [40–41] and induction of secondary structures [42–46]. Carbohydrate-derived β-amino acids used in this study were obtained by conjugate addition (Michael addition) of ammonia to a carbohydrate derived α,β-unsaturated ester [47–50].

**Figure 2:**
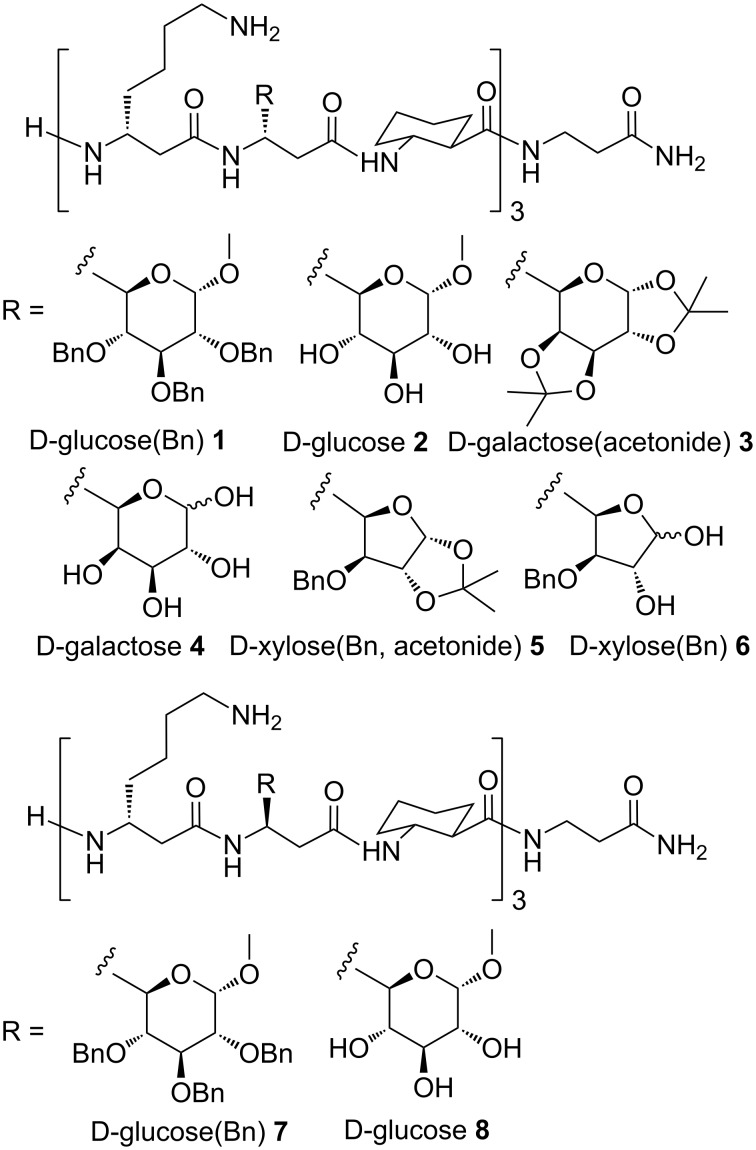
Synthesized β-glycopeptides **1**–**8**.

In the present article, a new class of C-linked β-glycopeptide scaffolds **1**–**8** (Figure 2) were synthesized and investigated with respect to secondary structures, along with the influence of glycan modifications on peptide conformation [24–25], and the potential of defined sugar presentation on a template. The β-glycopeptides are synthetically accessible by incorporation of sugar amino acids in β-peptide helical secondary structures, whereas sugar-β-amino acids were derived from a continuation of our efforts in synthesis of sugar-amino acids [51–52].

β^3^-Amino acids were used in SPPS in order to get a conformationally stable and well-defined β-peptide 14-helix. The helix propensity is further improved by incorporation of the constrained cyclic amino acid *trans*-(1*R*,2*R*)-2-aminocyclohexanecarboxylic acid (ACHC) [53]. β-Homolysine was used to assure the peptide solubility in aqueous solution. Therefore, the β-peptide design provides incorporation of D-glucose, D-galactose and D-xylose derived sugar-β-amino acids at every third position (*i* and *i*+3). The remaining positions were filled with β-homolysine and ACHC. For ease of synthesis, β-homoglycine amide was chosen as C-terminal amino acid. Further the C-glycosidic attachment of the sugar units at the peptide backbone was varied with respect to the configuration. As evident from CD spectroscopy, out of eight β-glycopeptides **1**–**8** (Figure 2), the five β-glycopeptides **2**–**6** were shown to adopt a 3_14_-helix conformation, thereby organizing the D-glucose, D-galactose or D-xylose carbohydrate epitopes on one face of the helix.

## Results and Discussion

### Synthesis of carbohydrate-β^3^-amino acids

**Scheme 1:**
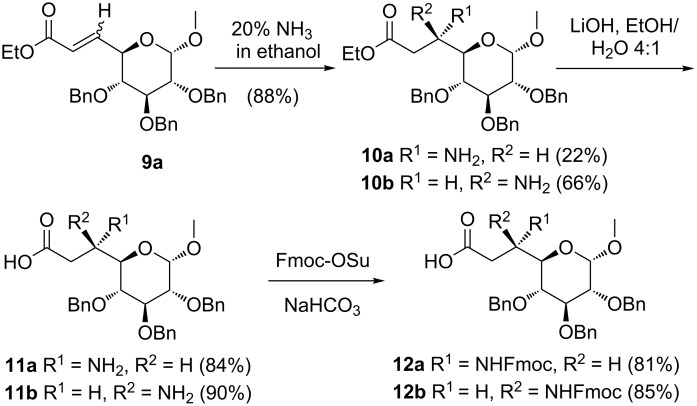
Synthesis of sugar-amino acid building blocks **12a** and **12b**.

Key step for the synthesis of β^3^-sugar amino acids is the Michael addition of ammonia to α,β-unsaturated esters [47–50]. Thus, preparation of the glucose derived β^3^-amino acid, the corresponding starting material ethyl (methyl 2,3,4-tri-*O*-benzyl-6,7-dideoxy-α-D-*gluco-*oct-6-enopyranoside)uronate (**9a**) was prepared from D-glucose as reported earlier [54]. Conjugate addition of ammonia to the α,β-unsaturated ester **9a** afforded a mixture of L- and D-*glycero* sugar-β-amino esters **10a** and **10b** in a 1:3 diastereomeric ratio as indicated by ^1^H NMR spectroscopy (Scheme 1).

**Figure 3:**
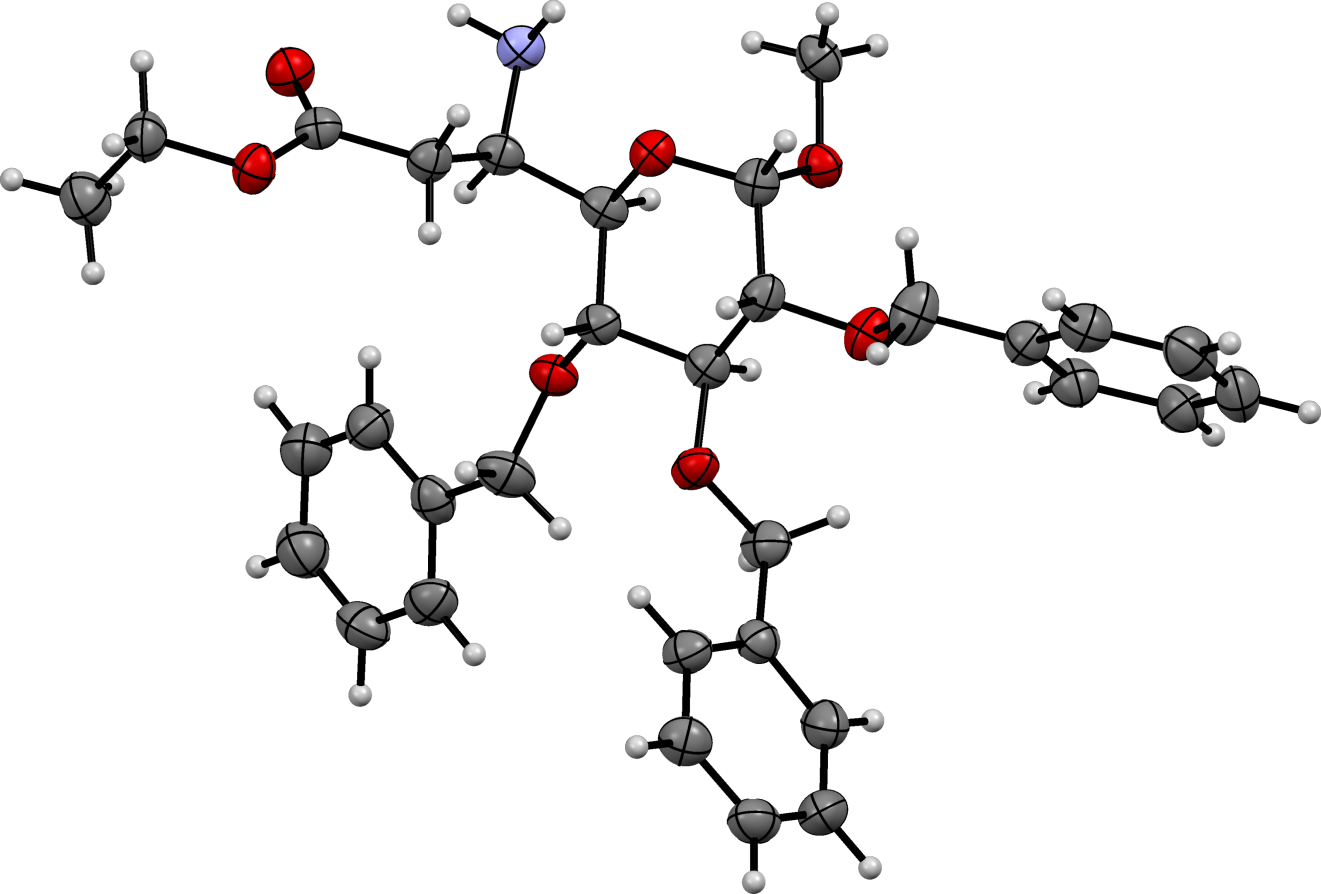
ORTEP diagram of compound **10b**.

**Figure 4:**
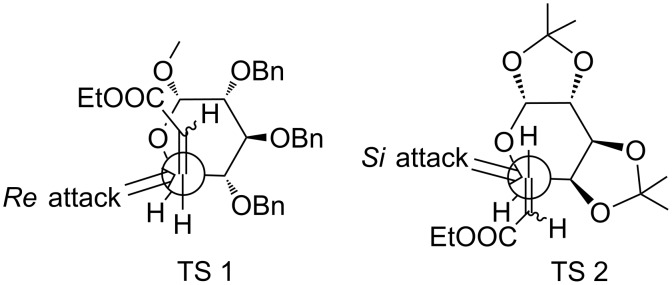
Preferential *re*-attack according to the Felkin–Anh model (TS 1) yielding **10b** (left) and *si*-attack (TS 2) providing **10c** (right).

The C6-epimers **10a** and **10b** were separated by column chromatography and obtained in 22% and 66% yield, respectively. The major isomer **10b** was crystallized and based on X-ray data, the 6*R* absolute configuration and the formation of the D-*glycero*-isomer were confirmed (Figure 3). Therefore, the minor isomer **10a** was assigned the 6*S* absolute configuration. The formation of major isomer **10b** can be explained based on the Felkin–Anh model. Thus, the ammonia nucleophile preferentially attacks from the *re*-face as shown in TS 1 (Figure 4) to give **10b** [48,55].

In the next step, ester saponification of **10a** using lithium hydroxide afforded D-glucose derived β-amino acid **11a** in 84% yield. Finally, *N*-terminal fluorenylmethoxycarbonyl (Fmoc) protection of the β-amino acid using Fmoc *N*-hydroxysuccinimidyl carbonate (Fmoc-OSu) under basic conditions gave sugar β-amino acid Fmoc-L-*glycero*-glucose(Bn)-OH **12a** in 81% yield (Scheme 1). The C6-epimer Fmoc-D-*glycero*-glucose(Bn)-OH **12b** was obtained by analogous procedures as performed for **12a**.

**Scheme 2:**
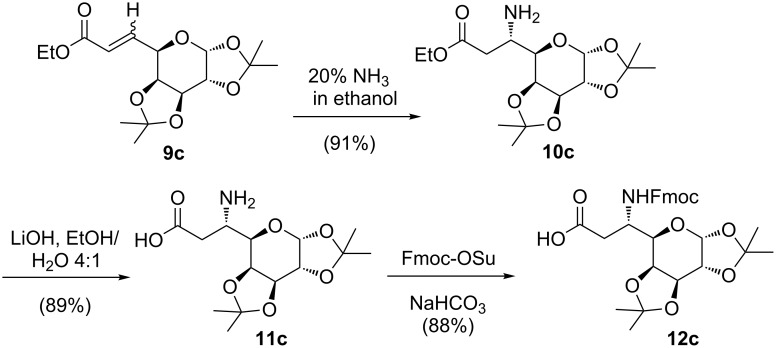
Synthesis of the galactosyl β-amino acid building block **12c**.

The synthesis of Fmoc-D-*glycero*-galactose-OH **12c** was carried out in analogy to the preparation of **12a** (Scheme 2). Thus, Michael addition of ammonia to D-galactose derived α,β-unsaturated ester **9c** [56] afforded **10c** (L-*glycero*) as a single diastereomer in 91% yield that was identified by spectral data identical to the reported [57]. Formation of compound **10c** is in accordance with the Felkin–Anh model. In case of compound **9c,** the TS 2 (Figure 4) is favourable with the attack of ammonia to the C=C double bond from the *si*-face affording compound **10c** as the only product.

Ester hydrolysis with lithium hydroxide followed by primary amine protection using Fmoc-OSu afforded the required scaffold Fmoc-L-glycero-galactose-OH **12c** (Scheme 2). The xylose derived *N*-Fmoc protected β-amino acid 1,2-*O*-isopropylidene-3-*O*-benzyl-5-deoxy-5-(*N*-9-fluorenylmethoxycarbonylamino)-β-L-*ido*-heptofuranuronic acid (Fmoc-L-*ido*-xylose-OH **12d**) was prepared following a literature protocol [49].

### Synthesis of β-glycopeptides **1–8**

Fmoc-protected sugar β-amino acids **12a–d** were found to be compatible with SPPS conditions. Therefore, the sugar units were introduced in the β-peptide like regular amino acids using a modified Fmoc SPPS protocol on mild acid sensitive Sieber amide resin. Simultaneous *tert*-butyloxycarbonyl (Boc)-deprotection and cleavage of the β-glycopeptides from solid support using 5% trifluoroacetic acid (TFA) in dry dichloromethane provided β-glycopeptides D-glucose(Bn) **1** and **7**, D-galactose(acetonide) **3**, and D-xylose(Bn, acetonide) **5** with all sugar hydroxy groups still being protected (Figure 2). Further, oligomers **1** and **7** were debenzylated using H_2_, 10% Pd/C to β-glycopeptides **2** and **8**, respectively. The acetonide groups in the galactosyl and xylosyl sugar units were deprotected simultaneously along with the Boc-groups and cleavage from solid support using TFA/water (4:1) [58] provided D-galactose and D-xylose derived β-glycopeptides **4** and **6**. The structural integrity of β-peptides **1**–**8** was ensured by high resolution ESI mass spectrometry.

### Conformational studies: carbohydrate influence on helical content

**Figure 5:**
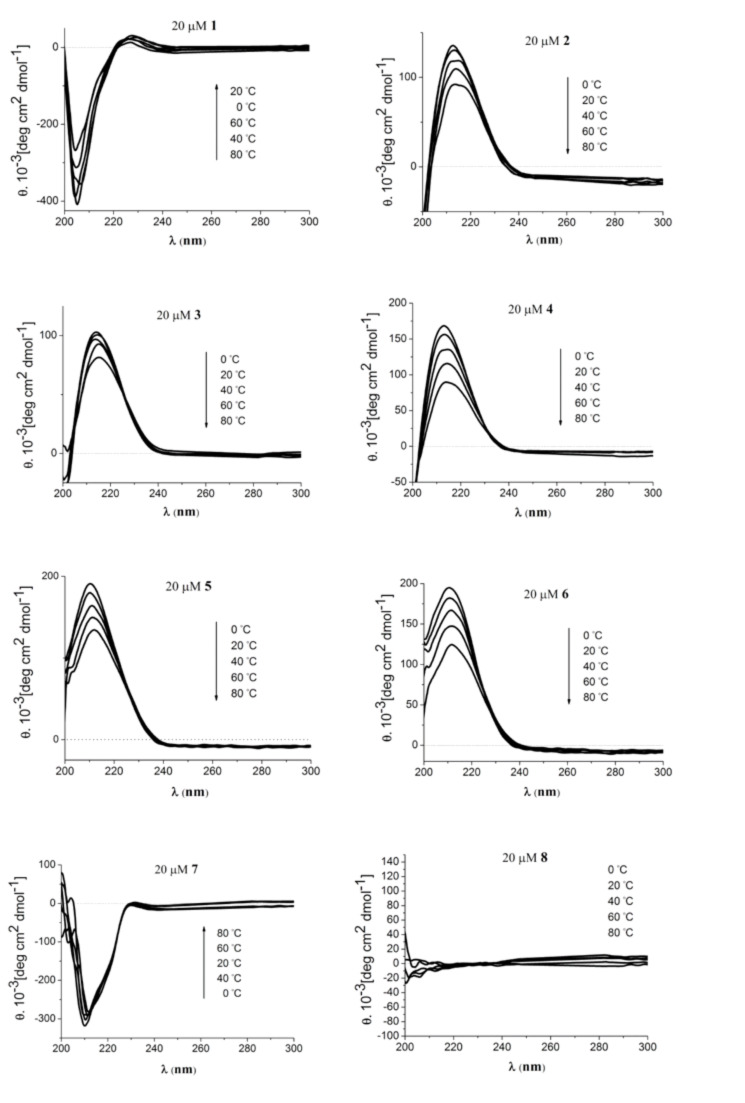
CD spectra of β-glycopeptides **1**–**8** (*c* = 20 μM) in triethylammonium acetate buffer (5 mM, pH 7) at various temperatures.

β-Peptides can be characterized by CD spectroscopy providing a positive Cotton effect at 215 nm for right-handed β^3^*-*peptides with 3_14_-helix conformation. The helical content correlates with the signal intensity and can be enhanced especially by ACHC incorporation at positions *i* and *i*+3; it is hardly sensitive to pH and ionic strength [59]. The CD spectra of the β-glycopeptides **1**–**8** were measured at pH 7 in triethylammonium acetate buffer at various temperatures (Figure 5). A strong positive Cotton effect at 215 nm for β-glycopeptides **2**–**6** indicates a right-handed 3_14_-helix conformation [4–6]. Even at 80 °C the helical content drops only about 20–40% depending on the kind of sugar units incorporated. Oligomers **1**–**6** all have in common the (*S*)-configuration for the β^3^-sugar side chains; this is in agreement with the right-handed 3_14_-helix and a positive Cotton effect.

Nevertheless, β^3^*-*peptide **1** seems to provide a different structure and helical sense indicated by a negative and shifted (204 nm) Cotton signal. This is likely due to the triple benzyl protection on the sugar units and the resulting high sterical demand in combination with hydrophobicity [60]. A similar effect was noticed for β^3^*-*peptide **7** that differs from oligomer **1** only in the β^3^-sugar side chain configuration. The deprotected oligomer **8** having the (6*R*)-configuration of sugar-β-amino acids is not structured as expected for β-peptides with mixed β^3^-side chain configurations. Overall, there is a indication for the influence of the benzyl groups in β^3^*-*peptides **1** and **7** on the secondary structure.

In addition to the influence of side chain configuration and sterically demanding sugar protecting groups on the β-peptide helical content, a difference between glucose and galactose containing peptides **2** and **4** was noticed by an increase in signal intensity in case of the galactose **4** substitution. For the galactosyl β-peptides, the hemiacetal form of the sugar units might be in equilibrium with the aldehyde form. The non-protected sugar units **4** seem to adopt better to the 3_14_-helix conformation. Nevertheless, the galactosyl β-peptide **3** with 1,2-3,4-acetonide protection also seems sterically not demanding compared to the benzyl groups. Similarly, the five-membered xylose derivatives **5** and **6** fit nicely into the 3_14_-helix structure and even the benzyl and isopropylidene protecting groups do not affect the β-peptide conformation.

## Conclusion

The Michael addition strategy was explored for the synthesis of sugar Fmoc-β^3^-amino acids building blocks that were further introduced in β-peptide sequences generating a new class of functionalized C-linked β-glycopeptides **1**–**8**. This kind of architecture allows the presentation of carbohydrate epitopes at defined distances on the same side of the β-peptide 3_14_-helix. The sugar hydroxy groups permit additional interactions of the water-soluble peptides. Further, uniform orientation and defined distances of side-chain sugar residues offer the opportunity to use sugar functionalized peptide scaffolds to study multivalency in carbohydrate recognition. The glucose, galactose and xylose derivatives were incorporated as sugar units in rigid peptide templates. Keeping the proper β^3^-configuration for getting a β-peptide 3_14_-helix (**2**–**6**) the isopropylidene protecting group and an anomeric acetal are structurally well tolerated. An additional perspective emerged from the unprotected β-glycopeptides since the equilibrium between hemiacetal and open-chain aldehyde form also does not interfere with the 3_14_-helix secondary structure and allows further functionalization with saccharides, e.g., by reductive amination.

## Supporting Information

File 1 Experimental section and copies of ^1^H and ^13^C NMR spectra of compounds **10a, 10b, 11a, 11b, 11c, 12a, 12b** and **12c**, HPLC traces of purified β-glycopeptides **1**–**8** as well as crystallographic data of compound **10b**.
