# Supplementary material for: Molecular architecture with carbohydrate functionalized β-peptides adopting 314-helical conformation
Source: Beilstein J Org Chem. 2014 Apr 28;10:948–55. doi: 10.3762/bjoc.10.93 (PMC4077383; doi:10.3762/bjoc.10.93)
Supplement: File 1 — Experimental section and copies of 1H and 13C NMR spectra of compounds 10a, 10b, 11a, 11b, 11c, 12a, 12b and 12c, HPLC traces of purified β-glycopeptides 1–8 as well as crystallographic data of compound 10b. [file Beilstein_J_Org_Chem-10-948-s001.pdf]

# Supporting Information

## for

# Molecular architecture with carbohydrate functionalized β-peptides adopting $3_{14}$-helical conformation

Nitin J. Pawar[1,2], Navdeep S. Sidhu[3], George M. Sheldrick[3], Dilip D. Dhavale*[2] and Ulf Diederichsen*[1]

Address: [1]Institute for Organic and Biomolecular Chemistry, Georg-August University Göttingen, Tammannstrasse 2, D-37077 Göttingen, Germany, [2]Department of Chemistry, Garware Research Centre, University of Pune, Pune 411 007, India and [3]Institute for Inorganic Chemistry, Georg-August University Göttingen, Tammannstraße 4, D-37077 Göttingen, Germany

Email: Ulf Diederichsen* - udieder@gwdg.de; Dilip D. Dhavale* - ddd@chem.unipune.ac.in

* Corresponding author

**Experimental section and copies of $^{1}$H and $^{13}$C NMR spectra of compounds 10a, 10b, 11a, 11b, 11c, 12a, 12b and 12c, HPLC traces of purified β-glycopeptides 1–8 as well as crystallographic data of compound 10b**

# 1. Experimental

## General remarks

Starting materials and reagents were purchased from *Sigma Aldrich*, *TCI*, *Fluka* and used as received. *N*-Methyl-2-pyrrolidone (NMP) for oligomer synthesis was obtained from *Carl Roth GmbH* and used without additional purification. Fmoc-(*R*,*R*)-ACHC-OH was prepared as described in literature [S1]. Fmoc-β-HLys(Z)-OH was obtained by the Arndt–Eistert homologation of the respective α-amino acid [S2]. Fmoc-Sieber amide resin for SPPS was purchased from *Novabiochem* (*Merck*) and stored at 4 °C. The 20% ammonia solution in ethanol was prepared by condensing 10 mL ammonia in 40 mL EtOH at −40 °C. Before opening, chemicals were warmed to room temperature. Analytical thin-layer chromatography (TLC) was performed on silica gel precoated aluminum sheets from *Merck* (silica gel 60-$F_{254}$, layer thickness 0.25 mm) and detected under UV lamp or by developing with 2,4-dinitrophenylhydrazine (2,4-DNP) solution or $I_2$. Flash column chromatography was performed on *Merck* silica gel 60 (40–60 µm) at 0.3 to 1.1 bar pressure. The solvents for flash column chromatography were distilled prior to use. For HPLC analysis $CH_3CN$ (HPLC grade) and ultra pure $H_2O$ (*Millipore*, Bedford, UK) were used. Melting points were obtained with a Bibby-SMP10 melting point apparatus and are uncorrected. Optical rotations were measured on a Perkin–Elmer 241 polarimeter. IR spectra were recorded on a Digilab Excalibur FTIR spectrometer. $^1$H and $^{13}$C NMR spectra were recorded at 298 K in deuterated solvents using Bruker Avance 300/500 MHz spectrometers. Mass spectra (ESI–MS) were recorded on a Finnigan LCQ mass spectrometer. High-resolution mass spectra (HRMS–ESI) were obtained with the Bruker Apex-Q IV FT-ICR mass spectrometer. CD spectra were recorded on a JASCO J-810 spectrometer equipped with a JASCO ETC-505S/PTC-423S temperature controller. HPLC analysis

and purification of the oligomers was performed on a Pharmacia Äkta Basic system (GE Healthcare, London, UK) with a pump type P-900, variable wavelength detector UV-900 using C18 MN-Nucleodur 100-5 C-18 (250 × 4.6 mm, 5 μm) analytical HPLC column. All HPLC runs were performed by using a linear gradient of A (0.1% aq TFA) and B (80% aq $CH_3CN$ and 0.1% TFA). Flow rates were taken as 1 mL $min^{-1}$ for the analytical HPLC. For HPLC, glycoconjugates were dissolved in Milli Q $H_2O$. All sample solutions were filtered prior to injection. UV detection was conducted at 215 nm. All CD measurements were carried out at 20 μM peptide concentration in 5 mM triethylammonium acetate buffer (pH 7.0) with a quartz cell of 1 cm path length. Spectra represent the average of 6 scans after baseline correction.

## Synthesis of compounds

**Ethyl (methyl 2,3,4-tri-*O*-benzyl-6-amino-6,7-dideoxy-L-*glycero*-β-D-*gluco*-octo-pyranoside)uronate (10a) and ethyl (methyl 2,3,4-tri-*O*-benzyl-6-amino-6,7-dideoxy-D-*glycero*-α-D-*gluco*-octo-pyranoside)uronate (10b).** A solution of compound **9a** (4.0 g, 7.51 mmol) in 20% ammonia in ethanol (50 mL) was stirred at 23 °C for 24 h in a sealed tube. Solvent evaporation was followed by chromatographic purification. First elution using $CH_2Cl_2$/MeOH, 99:1 gave the minor isomer (**10a**) (0.90 g, 22%) as viscous liquid; $R_f$ = 0.55 ($CH_2Cl_2$/MeOH 95:5); $[\alpha]_D^{23}$ = +46.4 (c, 0.22, MeOH); IR (neat, ν, $cm^{-1}$): 2900-3200, (br), 1717; $^1H$ NMR (300 MHz, $CDCl_3$) δ 1.24 (t, $J$ = 7.1 Hz 3H, $CH_3$), 1.30 (s, exchangeable with $D_2O$, 2H, $NH_2$), 2.40 (dd, $J$ = 15.6, 4.7 Hz, 1H, H-7), 2.51 (dd, $J$ = 15.6, 8.9 Hz, 1H, H-7') 3.32 (s, 3H, $CH_3$), 3.40-3.55 (m, 3H, H-2, H-5, H-6), 3.66 (t, $J$ = 9.0 Hz, 1H, H-4), 3.98 (t, $J$ = 9.0 Hz, 1H, H-3), 4.13 (q, $J$ = 7.1 Hz, 2H, $CH_2CH_3$), 4.54 (d, $J$ = 3.6 Hz, 1H, H-1), 4.64 (d, $J$ = 12.0 Hz, 1H, $CH_2Ph$), 4.71 (d, $J$ = 11.0 Hz, 1H, $CH_2Ph$), 4.78 (d, $J$ = 12.0 Hz, 1H,

$CH_2Ph$), 4.82 (d, *J* = 10.9 Hz, 1H, $CH_2Ph$), 4.89 (d, *J* = 11.0 Hz, 1H, $CH_2Ph$), 4.98 (d, *J* = 10.9 Hz, 1H, $CH_2Ph$), 7.26-7.35 (m, 15H, $H_{Ar}$); $^{13}C$ NMR (125 MHz, $CDCl_3$) δ 14.3, 41.0, 47.0, 55.2, 60.5, 72.5, 73.4, 74.8, 75.6, 77.1, 79.9, 82.2, 98.2, 127.5, 127.7, 127.8, 127.9, 128.0, 128.1, 128.3, 128.4, 138.1, 138.2, 138.8, 172.1 (C=O); MS (ESI): *m*/*z* 550.3 $[M+H]^+$; HR-MS (ESI): *m*/*z* calcd. $C_{32}H_{39}NO_7$ $[M+H]^+$: 550.2799, found: 550.2801. calcd. $[M-H]^-$: 548.2654, found: 548.2643. Further elution with $CH_2Cl_2$/MeOH, 99:1 to 95:5 provided **10b** (2.75 g, 66%) as crystalline colorless solid. $R_f$ = 0.44 ($CH_2Cl_2$/MeOH 95:5); mp 90–92 °C; $[\alpha]_D^{22}$ = +80.4 (c, 0.27, MeOH); IR (neat, ν, $cm^{-1}$): 2900–3200, (br), 1717; $^1H$ NMR (300 MHz, $CDCl_3$) δ 1.22 (t, *J* = 7.1 Hz 3H, $CH_3$), 1.57 (s, exchangeable with $D_2O$, 2H, $NH_2$), 2.24 (dd, *J* = 15.9, 9.0 Hz, 1H, H-7), 2.32 (dd, *J* = 15.9, 4.1 Hz, 1H, H-7’), 3.31 (dd, *J* = 10.0, 9.0 Hz, 1H, H-4) 3.36 (s, 3H, $CH_3$), 3.44 (dd, *J* = 9.0, 3.6 Hz, 1H, H-2), 3.50 (m, 1H, H-6), 3.66 (dd, *J* = 10.0, 2.8 Hz, 1H, H-5), 3.99 (t, *J* = 9.0 Hz, 1H, H-3), 4.10 (q, *J* = 7.1 Hz, 2H, $CH_2CH_3$), 4.52 (d, *J* = 3.6 Hz, 1H, H-1), 4.63 (d, *J* = 10.9 Hz, 1H, $CH_2Ph$), 4.64 (d, *J* = 12.1 Hz, 1H, $CH_2Ph$), 4.77 (d, *J* = 12.1 Hz, 1H, $CH_2Ph$), 4.80 (d, *J* = 10.8 Hz, 1H, $CH_2Ph$), 4.89 (d, *J* = 10.9 Hz, 1H, $CH_2Ph$), 4.99 (d, *J* = 10.8 Hz, 1H, $CH_2Ph$), 7.30-7.40 (m, 15H, $H_{Ar}$); $^{13}C$ NMR (125 MHz, $CDCl_3$) δ 14.2, 37.0, 48.5, 55.2, 60.4, 73.3, 73.5, 74.6, 75.7, 78.5, 80.1, 82.3, 97.7, 127.5, 127.7, 127.8, 127.9, 128.0, 128.1, 128.3, 128.4, 137.7, 137.9, 138.5, 172.4 (C=O); MS (ESI): *m*/*z* 550.3 $[M+H]^+$; HR-MS (ESI): *m*/*z* calcd. $C_{32}H_{39}NO_7$ $[M+H]^+$: 550.2799, found: 550.2801. calcd. $[M-H]^-$: 548.2654, found: 548.2648.

**Methyl 2,3,4-tri-*O*-benzyl-6-amino-6,7-dideoxy-L-*glycero*-β-D-*gluco*-octopyranuronic acid (11a).** To an ice-cooled solution of **10a** (0.85 g, 1.55 mmol) in EtOH/water (20 mL, 4/1), lithium hydroxide monohydrate (0.25 g, 6.25 mmol) was added, at 0 °C. The mixture was brought to 25 °C and the pH of the solution was adjusted to 7 by adding 0.5 M $H_3PO_4$. The solvent was evaporated, and the residue

was extracted with $CH_2Cl_2$ (40 mL 3×). Purification by column chromatography ($CH_2Cl_2$/MeOH 9/1) afforded **11a** (0.68 g, 84%) as colorless solid. $R_f$ = 0.47 ($CH_2Cl_2$/MeOH /25% aq $NH_4OH$ 89:10:1); mp 173-175 °C; $[\alpha]_D^{22}$ = +33.3 (c, 0.24, MeOH); IR (neat, v, $cm^{-1}$): 2900-3300, (br), 1700; $^1H$ NMR (300 MHz, $CDCl_3$ + $D_2O$) δ 2.19 (dd, *J* = 16.0, 2.1 Hz, 1H, H-7), 2.49 (dd, *J* = 16.0, 12.3 Hz, 1H, H-7'), 3.25 (s, 3H, $CH_3$), 3.35-3.55 (m, 4H, H-2, H-4, H5, H-6), 3.94 (t, *J* = 9.0 Hz, 1H, H-3), 4.55 (d, *J* = 3.3 Hz, 1H, H-1), 4.56 (d, *J* = 12.0 Hz, 1H, $CH_2Ph$), 4.67 (d, *J* = 11.1 Hz, 1H, $CH_2Ph$), 4.69 (d, *J* = 12.0 Hz, 1H, $CH_2Ph$), 4.74 (d, *J* = 11.1 Hz, 1H, $CH_2Ph$), 4.83 (d, *J* = 11.1 Hz, 1H, $CH_2Ph$), 4.95 (d, *J* = 11.1 Hz, 1H, $CH_2Ph$), 7.12-7.37 (m, 15H, $H_{Ar}$); $^{13}C$ NMR (125 MHz, $CDCl_3$ + $D_2O$) δ 37.0, 47.1, 55.4, 70.2, 73.3, 74.2, 75.5, 75.7, 79.8, 82.1, 97.9, 127.6, 127.8, 127.9, 128.1, 128.3, 128.4, 128.5, 137.6, 137.8, 138.2, 174.6 (C=O); MS (ESI): *m*/*z* 522.3 $[M+H]^+$; HR-MS (ESI): *m*/*z* calcd. $C_{30}H_{35}NO_7$ $[M+H]^+$: 522.2486, found: 522.2485. calcd. $[M-H]^+$: 520.2341, found: 520.2338. calcd. $[M+Na]^-$: 544.2306, found: 544.2306.

**Methyl 2,3,4-tri-*O*-benzyl-6-amino-6,7-dideoxy-D-*glycero*-α-D-*gluco*-octopyranuronic acid (11b).** In analogy to the synthesis of **11a**, the reaction of **10b** (2.00 g, 3.64 mmol) with lithium hydroxide monohydrate gave **11b** (1.70 g, 90%) as colorless solid. $R_f$ = 0.42 ($CH_2Cl_2$/MeOH/25% aq $NH_4OH$ 89:10:1); mp 192-194 °C; $[\alpha]_D^{22}$ = +103.5 (c, 0.39, MeOH); IR (neat, v, $cm^{-1}$): 2900-3200, (br), 1700; $^1H$ NMR (300 MHz, $CDCl_3$ + $D_2O$) δ 2.09 (dd, *J* = 16.8, 3.0 Hz, 1H, H-7), 2.32 (dd, *J* = 16.8, 11.1 Hz, 1H, H-7'), 3.25 (dd, *J* = 10.2, 9.0 Hz, 1H, H-4), 3.30 (s, 3H, $CH_3$), 3.40 (dd, *J* = 9.0, 3.3 Hz, 1H, $H_2$), 3.63 (dt, *J* = 11.1, 3.0 Hz, 1H, H-6), 3.80 (dd, *J* = 10.2, 3.0 Hz, 1H, H-5), 3.92 (t, *J* = 9.0 Hz, 1H, H-3), 4.53-4.62 (m, 3H, H-1, $CH_2Ph$), 4.66 (d, *J* = 12.0 Hz, 1H, $CH_2Ph$), 4.68 (d, *J* = 10.8 Hz, 1H, $CH_2Ph$), 4.76 (d, *J* = 11.1 Hz, 1H, $CH_2Ph$), 4.93 (d, *J* = 10.8 Hz, 1H, $CH_2Ph$), 7.10-7.35 (m, 15H, $H_{Ar}$); $^{13}C$ NMR (125 MHz, $CDCl_3$ + $D_2O$) δ 33.1, 48.8, 55.9, 69.5, 73.2, 74.4, 75.6, 77.0, 79.9, 81.9, 97.9,

127.6, 127.7, 127.9, 128.1, 128.2, 128.3, 128.4 128.5, 137.2, 137.8, 138.3, 175.4 (C=O); MS (ESI): *m*/*z* 522.3 [M+H] $^{+}$; HR-MS (ESI): *m*/*z* calcd. $C_{30}H_{35}NO_7$ $[M+H]^+$: 522.2486, found: 522.2483. calcd. $[M+Na]^+$: 544.2306, found: 544.2300.

**Methyl 2,3,4-tri-*O*-benzyl-6-(*N*-9-fluorenylmethoxycarbonylamino)-6,7-dideoxy-L-*glycero*-β-D-*gluco*-octopyranuronic acid (12a).** To an ice-cooled solution of **11a** (0.60 g, 1.15 mmol) in 1,4-dioxane/$H_2O$, 2:1 (10 mL), Fmoc-OSu (0.43 g, 1.27 mmol) and $NaHCO_3$ (0.97 g, 11.5 mmol) were added. The turbid reaction mixture was stirred at 0 °C for 1 h and then stirred at room temperature overnight. The pH of the solution was adjusted to 7 by adding 0.5 M $H_3PO_4$, and extracted with ethyl acetate (40 mL, 3×). Purification by column chromatography ($CH_2Cl_2$/MeOH 99:1) afforded **12a** (0.69 g, 81%) as colorless solid. $R_f$ = 0.55 ($CH_2Cl_2$/MeOH 95:5); mp 70-72 °C; $[\alpha]_D^{22}$ = –1.2 (c, 0.44, MeOH); IR (neat, v, $cm^{-1}$): 2900-3300, (br), 1710, 1693,; $^1H$ NMR (300 MHz, $CDCl_3$ + $D_2O$) δ 2.50-2.60 (d, *J* = 6.9 Hz, 2H, H-7), 3.32 (s, 3H, $CH_3$), 3.33 (t, *J* = 9.6 Hz, 1H, H-4), 3.42 (dd, *J* = 9.6, 3.6 Hz, 1H, H-2), 3.66 (dd, *J* = 9.6, 1.2 Hz, 1H, H-5), 3.98 (t, *J* = 9.6, Hz, 1H, H-3), 4.17 (t, *J* = 6.6 Hz, 1H, CHFmoc), 4.37 (dd, *J* = 10.5, 6.6 Hz, 1H, $CH_2$CHFmoc), 4.49 (dd, *J* = 10.5, 6.6 Hz, 1H, $CH_2$CHFmoc), 4.55 (d, *J* = 3.6 Hz, 1H, H-1), 4.57-4.69 (m, 3H, H-6, $CH_2$Ph), 4.72-4.85 (m, 3H, $CH_2$Ph), 4.97 (d, *J* = 10.8 Hz, 1H, $CH_2$Ph), 7.12-7.39 (m, 19H, $H_{Ar}$), 7.55 (d, *J* = 7.2 Hz, 2H, Fmoc), 7.69-7.76 (m, 2H, Fmoc); $^{13}C$ NMR (125 MHz, $CDCl_3$) δ 37.5, 46.8, 47.3, 55.5, 66.6, 71.2, 73.4, 75.4, 75.8, 77.8, 79.7, 81.7, 98.1, 119.8, 120.9, 124.9, 125.0, 127.0, 127.1, 127.6, 127.7, 127.8, 127.9, 128.0, 128.1, 128.3, 128.4, 128.5, 137.9, 138.0, 138.5, 141.2, 143.6, 143.8, 155.7 (C=O), 174.0 (C=O); MS (ESI): *m*/*z* 744.6 $[M+H]^+$, 766.6 $[M+Na]^+$; HR-MS (ESI): *m*/*z* calcd. $C_{45}H_{45}NO_9$ $[M+H]^+$: 744.3167, found: 744.3149. calcd. $[M-H]^-$: 742.3022, found: 742.3012. calcd. $[M+Na]^+$: 766.2987, found: 766.2981.

**Methyl 2,3,4-tri-*O*-benzyl-6-(*N*-9-fluorenylmethoxycarbonylamino)-6,7-dideoxy-D-*glycero*-α-D-*gluco*-octopyranuronic acid (12b).** In analogy to the synthesis of **12a**, the reaction of **11b** (1.50 g, 2.88 mmol) with Fmoc-OSu yielded **12b** (1.81 g, 85%) as colorless solid. $R_f$ = 0.45 ($CH_2Cl_2$/MeOH 95:5); mp 177-178 °C; $[\alpha]_D^{22}$ = +31.7 (c, 0.51, MeOH); IR (neat, ν, $cm^{-1}$): 2900-3300, (br), 1710, 1695,; $^1H$ NMR (300 MHz, $CDCl_3$ + $D_2O$) δ 2.27 (m, 2H, H-7), 3.15-3.35 (m, 4H, H-4, $CH_3$), 3.43 (dd, *J* = 9.6, 3.6 Hz, 1H, H-2), 3.75 (dd, *J* = 10.2, 3.3 Hz, 1H, H-5), 3.97 (dd, *J* = 9.6, 8.4 Hz, 1H, H-3), 4.14 (t, *J* = 6.9 Hz, 1H, CHFmoc), 3.25-4.40 (m, 2H, $CH_2$CHFmoc), 4.45 (m, 1H, H-6), 4.50 (d, *J* = 3.6 Hz, 1H, H-1) 4.56-4.82 (m, 4H, $CH_2$Ph), 4.85 (d, *J* = 11.1 Hz, 1H, $CH_2$Ph), 4.97 (d, *J* = 10.8 Hz, 1H, $CH_2$Ph), 7.00-7.40 (m, 19H, $H_{Ar}$), 7.48-7.53 (m, 2H, Fmoc), 7.70 (d, *J* = 6.6 Hz, 2H, Fmoc); $^{13}C$ NMR (125 MHz, $CDCl_3$) δ 33.8, 47.1, 48.2, 55.1, 66.7, 71.7, 73.3, 74.2, 75.7, 77.6, 80.0, 82.1, 97.8, 119.9, 124.9, 125.0, 127.0, 127.6, 127.7, 127.8, 127.9, 128.0, 128.1, 128.3, 128.4, 128.5, 137.7, 137.9, 138.4, 141.2, 143.7, 143.9, 155.5 (C=O), 176.0 (C=O); MS (ESI): *m*/*z* 744.6 $[M+H]^+$, 766.6 $[M+Na]^+$; HR-MS (ESI): *m*/*z* calcd. $C_{45}H_{45}NO_9$ $[M+H]^+$: 744.3167, found: 744.3153. calcd. $[M+Na]^+$: 766.2987, found: 766.2988.

**Ethyl (methyl 1,2:3,4-di-*O*-isopropylidene-6-amino-6,7-dideoxy-L-*glycero*-β-D-*galacto*-octo-pyranoside)uronate (10c).** Reaction of **9c** (4.00 g, 12.18 mmol) with 20% ammonia in ethanol as described for **9a** gave **10c** (3.82 g, 91%) as viscous liquid. Rf 0.64 ($CH_2Cl_2$/MeOH 95:5); $[\alpha]_D^{22}$ = –64.7 (c, 0.18, $CHCl_3$); IR (neat, ν, $cm^{-1}$): 3300-3500, (br), 1725; $^1H$ NMR (300 MHz, $CDCl_3$) δ 1.21 (t, *J* = 7.1 Hz 3H), 1.28 (s, 6H), 1.40 (s, 3H), 1.47 (s, 3H), 1.70 (bs, exchangeable with $D_2O$, 2H), 2.41 (dd, *J* = 16.1, 7.0 Hz, 1H), 2.61 (dd, *J* = 16.1, 4.5 Hz, 1H), 3.30-3.45 (m, 1H), 3.58 (dd, *J* = 7.0, 1.7 Hz, 1H), 4.10 (q, *J* = 7.1 Hz, 2H), 4.22 (dd, *J* = 7.9, 1.7 Hz, 1H), 4.27 (dd, *J* = 5.0, 2.2 Hz, 1H), 4.54 (dd, *J* = 7.9, 2.2 Hz, 1H), 5.52 (d, *J* = 5.0 Hz, 1H); $^{13}C$ NMR

(125 MHz, $CDCl_3$) δ 14.2, 24.2, 24.9, 25.8, 25.9, 37.8, 48.5, 60.3, 70.3, 70.5, 70.8, 71.5, 96.4, 108.5, 109.2, 172.0 MS (ESI) *m*/*z* : 346.2 $[M+H]^+$; HR-MS (ESI) *m*/*z* : calcd. $C_{16}H_{27}NO_7$ $[M+H]^+$: 346.1860, found: 346.1862. calcd. $[M-H]^-$: 344.1715, found: 344.1711.

**Methyl 1,2:3,4-di-*O*-isopropylidene-6-amino-6,7-dideoxy-L-*glycero*-β-D-*galacto*-octopyranuronic acid (11c).** In analogy to the synthesis of **11a**, the reaction of **10c** (1.62 g, 4.69 mmol) with lithium hydroxide monohydrate gave **11c** (1.32 g, 89%) as colorless solid. $R_f$ = 0.42 ($CH_2Cl_2$/MeOH/25% aq $NH_4OH$ 79:20:1); mp 220-222 °C; $[\alpha]_D^{22}$ = –72.2 (c, 0.27, MeOH); IR (neat, ν, $cm^{-1}$): 2900-3300, (br),1700; $^1H$ NMR (300 MHz, $CD_3OD$) δ 1.33 (s, 3H, $CH_3$), 1.35 (s, 3H, $CH_3$), 1.43 (s, 3H, $CH_3$), 1.52 (s, 3H, $CH_3$), 2.45 (dd, *J* = 17.0, 8.0 Hz, 1H, H-7), 2.59 (dd, *J* = 17.0, 4.4 Hz, 1H, H-7’), 3.58 (m, 1H, H-6), 3.95 (dd, *J* = 7.3, 1.8 Hz, 1H, H-5), 4.37(dd, *J* = 7.9, 1.8 Hz, 1H, H-4), 4.42 (dd, *J* = 5.0, 2.5 Hz, 1H, H-2), 4.69 (dd, *J* = 7.9, 2.5 Hz, 1H, H-3), 5.57 (d, *J* = 5.0 Hz, 1H, H-1); $^{13}C$ NMR (75 MHz, $CD_3OD$) δ 24.4, 25.1, 26.2, 26.3, 35.5, 51.3, 68.3, 71.8, 72.1, 72.2, 97.7, 110.4, 111.0, 176.9 (C=O); MS (ESI): *m*/*z* 318.2 $[M+H]^+$; HR-MS (ESI): *m*/*z* calcd. $C_{14}H_{23}NO_7$ $[M+H]^+$: 318.1547, found: 318.1549. calcd. $[M-H]^-$: 316.1402, found: 316.1404. calcd. $[M+Na]^+$: 340.1367, found: 340.1365.

**Methyl 1,2:3,4-di-*O*-isopropylidene-6-(*N*-9-fluorenylmethoxycarbonylamino)-6,7-dideoxy-L-*glycero*-β-D-*galacto*-octopyranuronic acid (12c).** In analogy to the synthesis of **12a**, the reaction of **11c** (1.00 g, 3.15 mmol) with Fmoc-OSu produced **12c** (1.49 g, 88%) as colorless solid. $R_f$ = 0.60 ($CH_2Cl_2$/MeOH 9:1); mp 73-75 °C; $[\alpha]_D^{22}$ = –35.9 (c, 0.67, MeOH); IR (neat, ν, $cm^{-1}$): 2900-3300, (br),1710, 1691; $^1H$ NMR (300 MHz, $CDCl_3$ + $D_2O$) δ 1.29 (s, 6H, 2 ($CH_3$)), 1.41 (s, 3H, $CH_3$), 1.48 (s, 3H, $CH_3$), 2.70-2.90 (m, 2H, H-7), 4.04 (m, 1H, H-5), 4.17-4.27 (m, 3H, H-4, H-6, CHFmoc), 4.29 (dd, *J* = 4.8, 2.4 Hz, 1H, H-2), 4.31-4.41 (m, 2H, $CH_2$CHFmoc), 4.57 (dd, *J* = 8.0, 2.4 Hz, 1H, H-3), 5.54 (d, *J* = 4.8 Hz, 1H, H-1), 7.20-740 (m, 4H, Fmoc),

7.50-765 (m, 2H, Fmoc), 7.70-780 (m, 2H, Fmoc); $^{13}C$ NMR (125 MHz, $CDCl_3$ + $D_2O$) δ 24.3, 25.0, 25.9, 26.0, 35.5, 47.2, 48.9, 66.7, 66.8, 70.6, 71.1, 71.7, 96.5, 108.8, 109.5, 119.8, 125.1, 126.9, 127.5, 141.1, 141.2, 143.8, 143.9, 156.2 (C=O), 176.1 (C=O); MS (ESI): *m*/*z* 540.3 $[M+H]^+$, 562.3 $[M+Na]^+$, 538.2 $[M-H]^+$; HR-MS (ESI): *m*/*z* calcd. $C_{29}H_{33}NO_9$ $[M+H]^+$: 540.2228, found: 540.2225. calcd. $[M-H]^-$: 538.2083, found: 538.2066. calcd. $[M+Na]^+$: 562.2048, found: 562.2044.

## General procedure for solid-phase β-glycopeptide synthesis

Oligomers were prepared in a similar manner as described in reference [S3] by manual solid-phase peptide synthesis in a 2 mL BD syringe, using a Fmoc-Sieber amide resin with a loading capacity of 0.61 mmol $g^{-1}$. For oligomer syntheses a resin preloaded with H-β-HGly-OH (40.0 mg resin, 25 µmol homoglycine amide) was used. For peptide bond formation double coupling of the amino acids at 50 °C was needed. First, an excess of 5 equivalents amino acid (125.0 µmol) was used, activated by O-(7-azabenzotriazol-1-yl)-*N*,*N*,*N*',*N*'-tetramethyluronium hexafluorophosphate (HATU; 42.7 mg, 112.5 µmol, 4.5 equiv), 1-hydroxy-7-azabenzotriazole (HOAt; 17.0 mg, 125.0 µmol, 5 equiv) and *N*,*N*-diisopropylethylamine (DIPEA; 60.9 µL, 350.0 µmol, 14 equiv) in NMP (400 µL); the second coupling was performed with 3 equivalents of amino acid (75.0 µmol) and activation with HATU (25.6 mg, 67.5 µmol, 2.7 equiv), HOAt (10.2 mg, 75.0 µmol, 3 equiv), and DIPEA (39.2 µL, 225.0 µmol, 9 equiv) in NMP (300 µL). After the swelling of the H-β-HGly-OH loaded resin for 2 h in $CH_2Cl_2$ (2 mL), the following procedure was carried out for each coupling step:

1. Fmoc-deprotection twice, 10 min with 20% piperidine in NMP (2 mL)
2. Washing four times with NMP (2 mL), then four times with $CH_2Cl_2$ (2 mL) and four times with NMP (2 mL)

3. Double coupling steps, each 1.5 h with gentle moving at 50 °C
4. Washing with NMP (3 × 2 mL), $CH_2Cl_2$ (3 × 2 mL), and NMP (3 × 2 mL);
5. Capping twice for 3 min with NMP/$Ac_2O$/DIPEA (8:1:1, 2 mL).
6. After the final coupling cycle, *N*-terminal Fmoc group was deprotected and resin washed with NMP (4 × 2 mL), DCM (4 × 2 mL), NMP (4 × 2 mL), MeOH (4 × 2 mL), DCM (4 × 2 mL) and dried overnight in vacuo.

**TFA cleavage and deprotection:** The glycopeptide species were selectively cleaved from the solid support under acidic conditions using 5% TFA in dry $CH_2Cl_2$. Cleavage reactions were carried out for 1 h, shaking at ambient temperature. The reaction mixture was neutralized by filtering it in 10% pyridine in $CH_2Cl_2$ and washing with MeOH. The resulting solution was concentrated under $N_2$ stream. The crude oligomer was isolated by precipitation from cold diethyl ether (−15 °C) and lyophilized. The crude peptide was dissolved in water/$CH_3CN$ and purified by HPLC. The crude oligomers **1** and **7** were directly subjected for benzyl deprotection using $H_2$, 10% Pd/C in MeOH (1 mL) for 48 h. The solutions were filtered off and washed with water/MeOH (1:1). Lyophilization was followed by HPLC purification affording glycopeptides **2** and **8**. The deprotection of acetonide groups of sugar (D-glucose, D-xylose), Boc-group of β-HLys and cleavage of peptide from the solid support were performed simultaneously using TFA/water (4:1). The cleavage reactions were carried out for 1 h shaking at ambient temperature. The solution was filtered and TFA was removed under $N_2$ stream, resulting solution were directly lyophilized. The crude peptides **4** and **6** were dissolved in water/acetonitrile and purified by HPLC.

**H-[β-HLys-β-(*S*)HAla(glucose(Bn))-ACHC]$_3$-HGly-NH$_2$ (1).** The cleavage reaction of the peptide was carried out by shaking the resin with 5% TFA in dry $CH_2Cl_2$ for 1 h at ambient temperature in a 2 mL BD syringe. The solution was neutralized by filtering in 10% pyridine in MeOH. The resulting solution was concentrated under $N_2$

stream. The crude oligomer was isolated by precipitation from cold diethyl ether (−15 °C) and dried. The crude peptide was dissolved in water/acetonitrile and purified by preparative HPLC (Nucleodur column, MN-C18, 250 × 4.6 mm, 5 μm, gradient 50–100% B2): $t_R$ = 27.2 min. ESI-MS: *m*/*z* = 801.2 $[M+3H]^{3+}$, 1201.2 $[M+2H]^{2+}$. HRMS (ESI): $C_{135}H_{182}N_{14}O_{25}$ *m*/*z* = 801.1230 $[M+3H]^{3+}$, calcd. 801.1217. 1201.1788 $[M+2H]^{2+}$, calcd. 1201.1789; 2401.3524 $[M+H]^{+}$, calcd. 2401.3505.

**H-[β-HLys-β-(*S*)HAla(glucose)-ACHC]$_3$-HGly-NH$_2$ (2).** Resin cleavage of the peptide was provided in analogy to **1** with 5% TFA giving crude glycopeptides **1** which was dissolved in MeOH and hydrogenated with $H_2$/10% Pd/C for 48 h. Filtration, solvent evaporation followed by HPLC purification using water/acetonitrile were performed to obtain β-glycopeptide **2**. HPLC (Nucleodur column, MN-C18, 250 × 4.6 mm, 5 μm, gradient 5–60% B2): $t_R$ = 23.3 min. ESI-MS: *m*/*z* = 530.7 $[M+3H]^{3+}$, 795.5 $[M+2H]^{2+}$, 1590.0 $[M+H]^{+}$. HRMS (ESI): $C_{72}H_{128}N_{14}O_{25}$ *m*/*z* = 530.6464 $[M+3H]^{3+}$, calcd. 530.6464, 795.4655 $[M+2H]^{2+}$, calcd. 795.4660.

**H-[β-HLys-β-(*S*)HAla(galactose(acetonide))-ACHC]$_3$-HGly-NH$_2$ (3).** Reaction of peptide loaded resin with 5% TFA as described for **1** produced β-glycopeptide **3**. HPLC (Nucleodur column, MN-C18, 250 × 4.6 mm, 5 μm, gradient 30–70% B2): $t_R$ = 21.6 min. ESI-MS: *m*/*z* = 597.0 $[M+3H]^{3+}$, 895.0 $[M+2H]^{2+}$, 1789.1 $[M+H]^{+}$. HRMS (ESI): $C_{87}H_{146}N_{14}O_{25}$ *m*/*z* = 597.0278 $[M+3H]^{3+}$, calcd. 597.0278, 895.0383 $[M+2H]^{2+}$, calcd. 895.0380.

**H-[β-HLys-β-(*S*)HAla(galactose)-ACHC]$_3$-HGly-NH$_2$ (4).** Cleavage of the peptide from the resin with a TFA/water (2 mL, 4:1) for 1 h at ambient temperature was followed by TFA evaporation under $N_2$ stream. After lyophilization, HPLC purification of crude product using water/acetonitrile provided β-glycopeptide **4**. HPLC (Nucleodur column, MN-C18, 250 × 4.6 mm, 5 μm, gradient 5–60% B2): $t_R$ = 20.0

min. ESI-MS: *m*/*z* = 516.6 $[M+3H]^{3+}$, 774.5 $[M+2H]^{2+}$, 1547.9 $[M+H]^{+}$. HRMS (ESI): $C_{69}H_{122}N_{14}O_{25}$ *m*/*z* = 516.6317 $[M+3H]^{3+}$, calcd. 516.6308, 774.4433 $[M+2H]^{2+}$, calcd. 774.4426, 1547.8786 $[M+H]^{+}$, calcd. 1547.8778, 1569.8601 $[M+Na]^{+}$, calcd. 1569.8598.

**H-[β-HLys-β-(*S*)HAla(xylose(Bn,acetonide))-ACHC]$_3$-HGly-NH$_2$ (5).** Reaction of peptide loaded resin with 5% TFA as described for **1** gave β-glycopeptide **5**. HPLC (Nucleodur column, MN-C18, 250 × 4.6 mm, 5 μm, gradient 40–90% B2): $t_R$ = 21.3 min. ESI-MS: *m*/*z* = 617.1 $[M+3H]^{3+}$, 925.1 $[M+2H]^{2+}$, 1849.2 $[M+H]^{+}$ . HRMS (ESI): $C_{96}H_{146}N_{14}O_{22}$ *m*/*z* = 617.0356 $[M+3H]^{3+}$, calcd. 617.0329, 925.0472 $[M+2H]^{2+}$, calcd. 925.0456, 1849.0818 $[M+H]^{+}$, calcd. 1849.0840, 1871.0661 $[M+Na]^{+}$, calcd. 1871.0659.

**H-[β-HLys-β-(*S*)HAla(xylose(Bn))-ACHC]$_3$-HGly-NH$_2$ (6).** Reaction of peptide loaded resin with 5% TFA as described for **4** afforded β-glycopeptide **6**. HPLC (Nucleodur column, MN-C18, 250 × 4.6 mm, 5 μm, gradient 25–80% B2): $t_R$ = 22.5 min. ESI-MS: *m*/*z* = 577.0 $[M+3H]^{3+}$, 865.0 $[M+2H]^{2+}$, 1729.1 $[M+H]^{+}$. HRMS (ESI): $C_{87}H_{134}N_{14}O_{22}$ *m*/*z* = 577.0021 $[M+3H]^{3+}$, calcd. 577.0016, 864.9990 $[M+2H]^{2+}$, calcd. 864.9988, 1728.9883 $[M+H]^{+}$, calcd. 1728.9903.

**H-[β-HLys-β-(*R*)HAla(glucose(Bn))-ACHC]$_3$-HGly-NH$_2$ (7).** Reaction of peptide loaded resin with 5% TFA as described for **1** gave β-glycopeptide **7**. HPLC (Nucleodur column, MN-C18, 250 × 4.6 mm, 5 μm, gradient 40–90% B2): $t_R$ = 26.7 min. ESI-MS: *m*/*z* = 801.1 $[M+3H]^{3+}$, 1201.2 $[M+2H]^{2+}$, 2401.3 $[M+H]^{+}$. HRMS (ESI): $C_{135}H_{182}N_{14}O_{25}$ *m*/*z* = 801.1218 $[M+3H]^{3+}$, calcd. 801.1217. 1201.1777 $[M+2H]^{2+}$, calcd. 1201.1789.

**H-[β-HLys-β-(*R*)HAla(glucose)-ACHC]$_3$-HGly-NH$_2$ (8).** Reaction of peptide loaded resin with 5% TFA followed by $H_2$, 10% Pd/C as described for **2** yielded β-

glycopeptide **8**. HPLC (Nucleodur column, MN-C18, 250 × 4.6 mm, 5 μm, gradient 5–40% B2): $t_R$ = 19.0 min. ESI-MS: *m*/*z* = 530.6 $[M+3H]^{3+}$, 795.5 $[M+2H]^{2+}$, 1589.9 $[M+H]^{+}$. HRMS (ESI): $C_{72}H_{128}N_{14}O_{25}$ *m*/*z* = 530.6465 $[M+3H]^{3+}$, calcd. 530.6464, 795.4664 $[M+2H]^{2+}$, calcd. 795.4660. 1589.9250 $[M+H]^{+}$, calcd. 1589.9248.

## 2. $^{1}H$ and $^{13}C$ NMR spectra of compounds 10a-b, 11a-c, and 12a-c

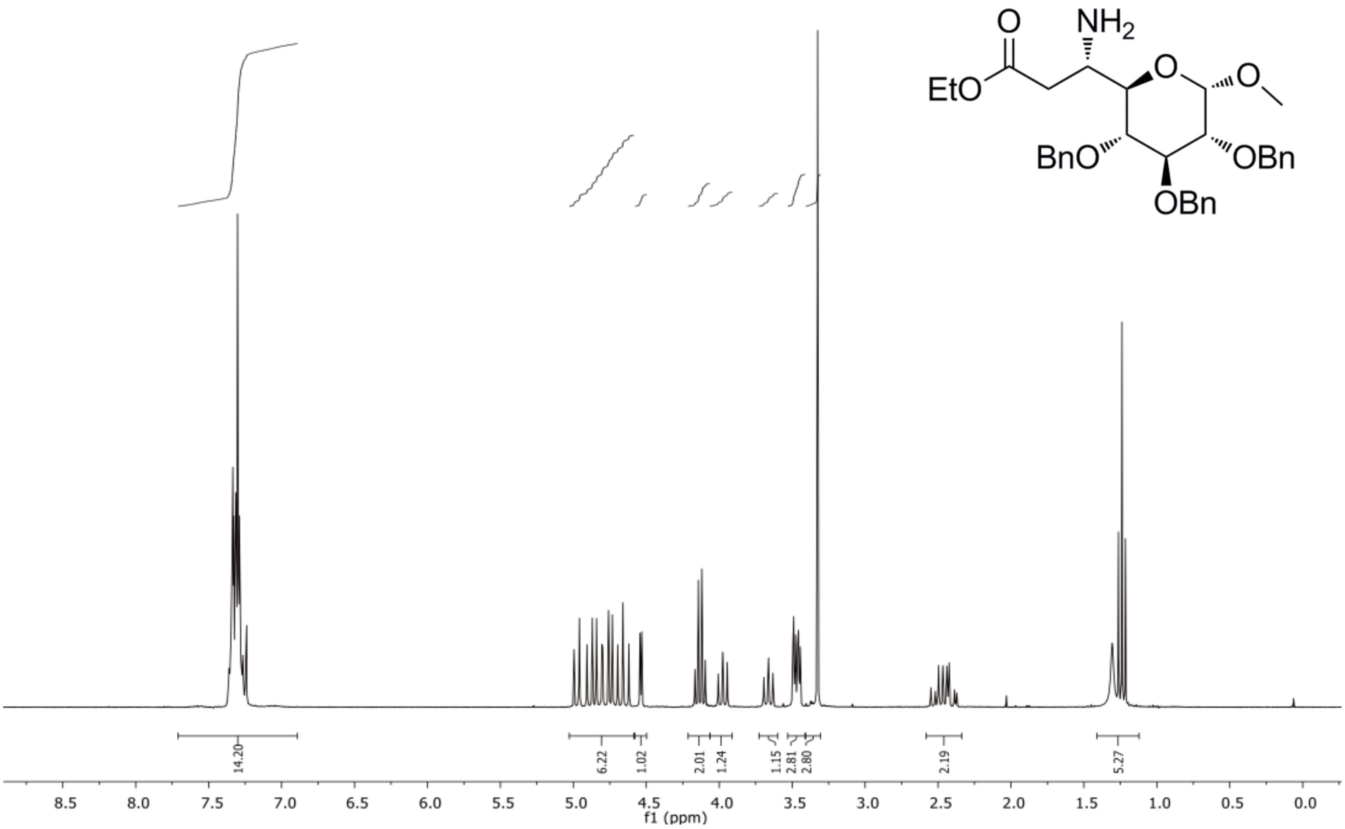


$^{1}H$ NMR (300 MHz, $CDCl_3$) spectrum of compound **10a**

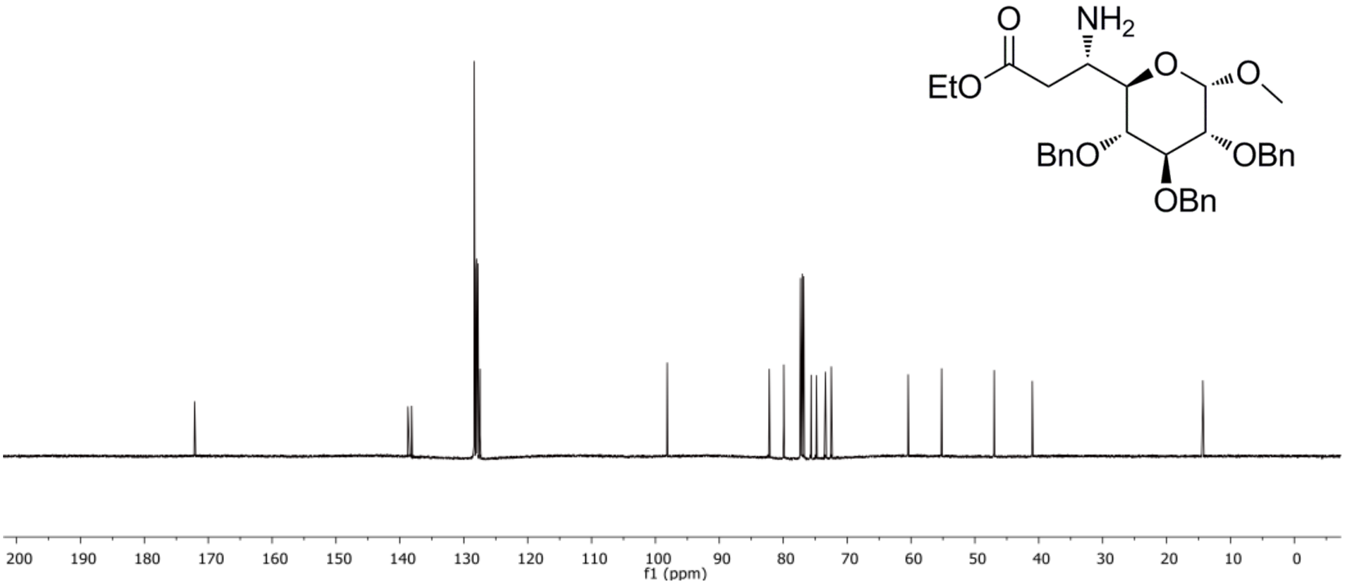


$^{13}C$ NMR (125 MHz, $CDCl_3$) spectrum of compound **10a**

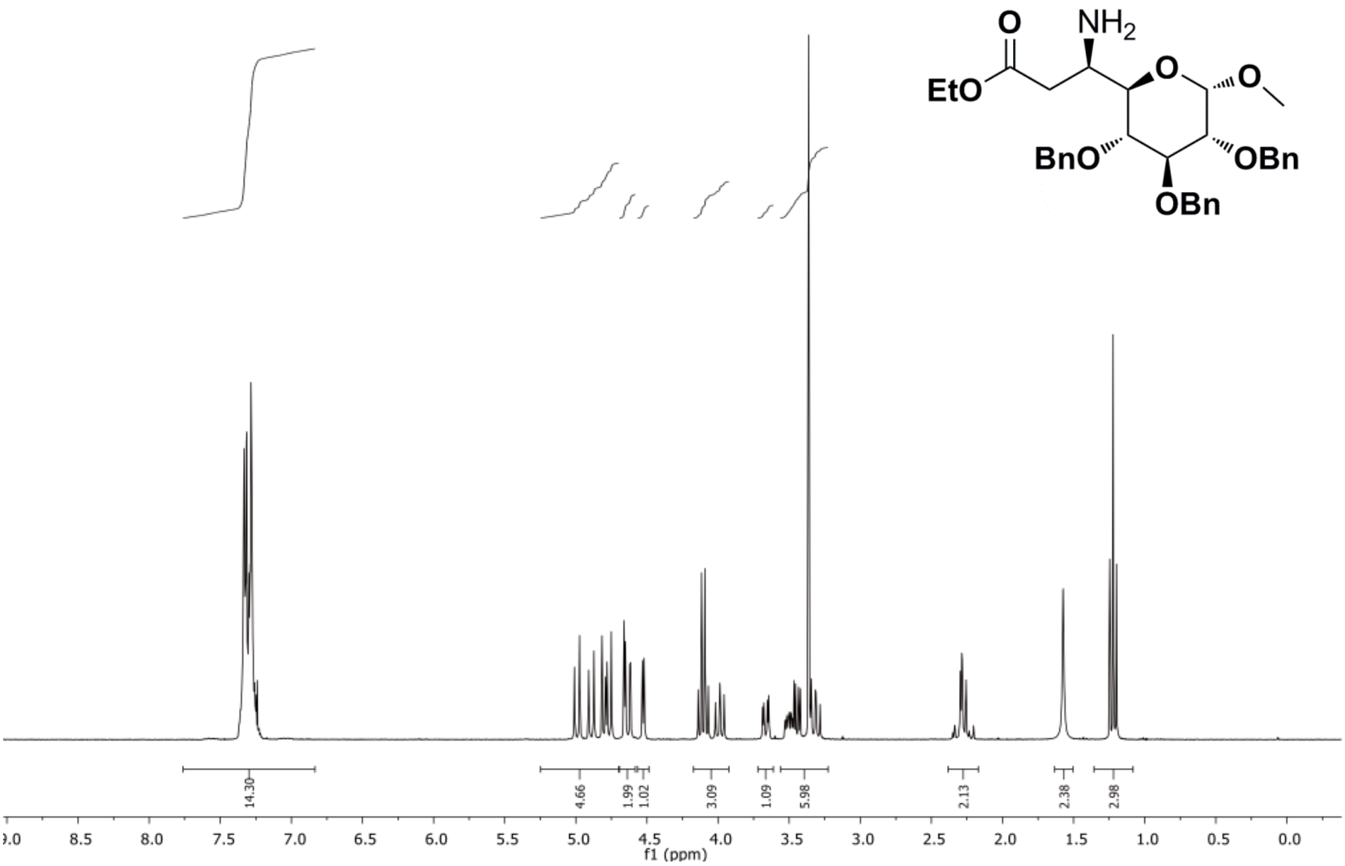


$^{1}H$ NMR (300 MHz, $CDCl_3$) spectrum of compound **10b**

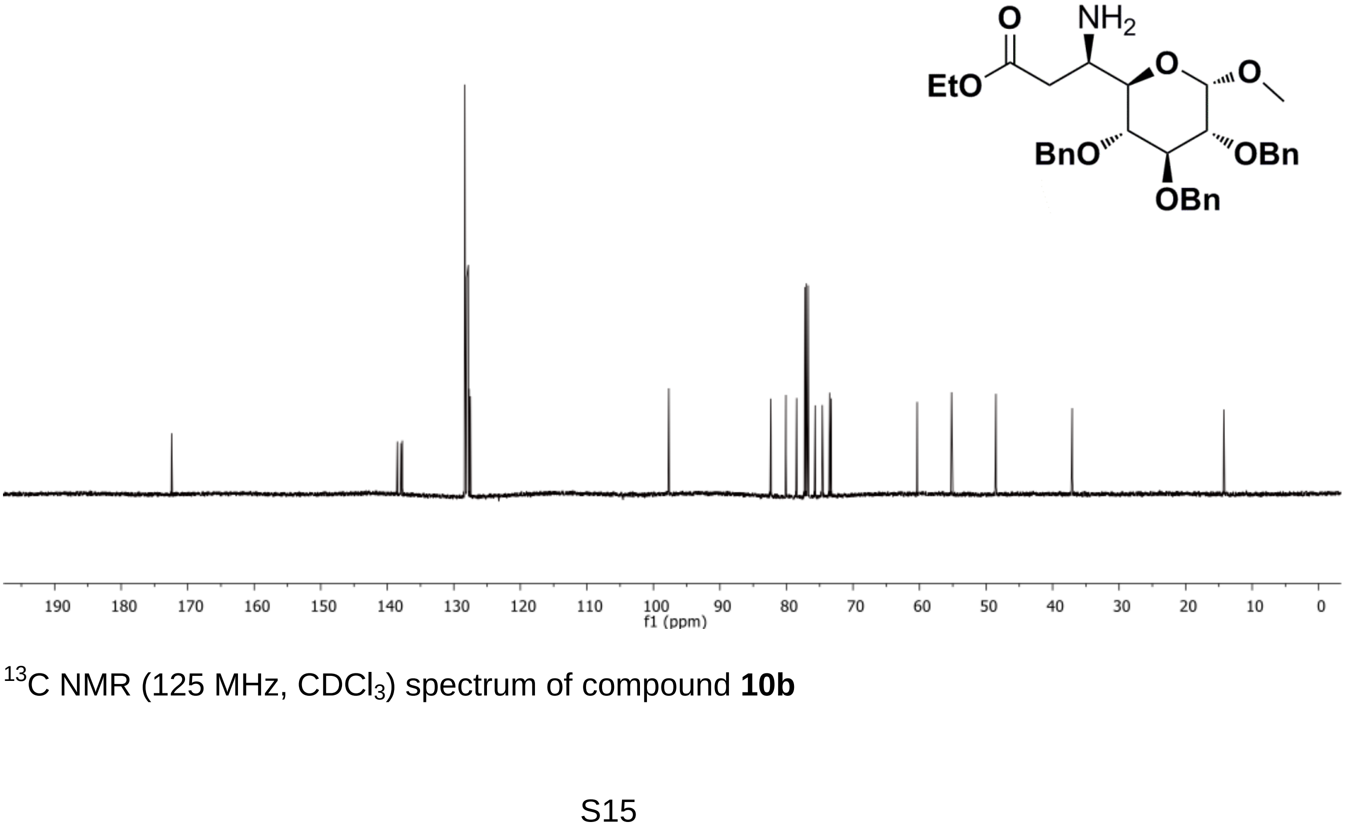


$^{13}C$ NMR (125 MHz, $CDCl_3$) spectrum of compound **10b**

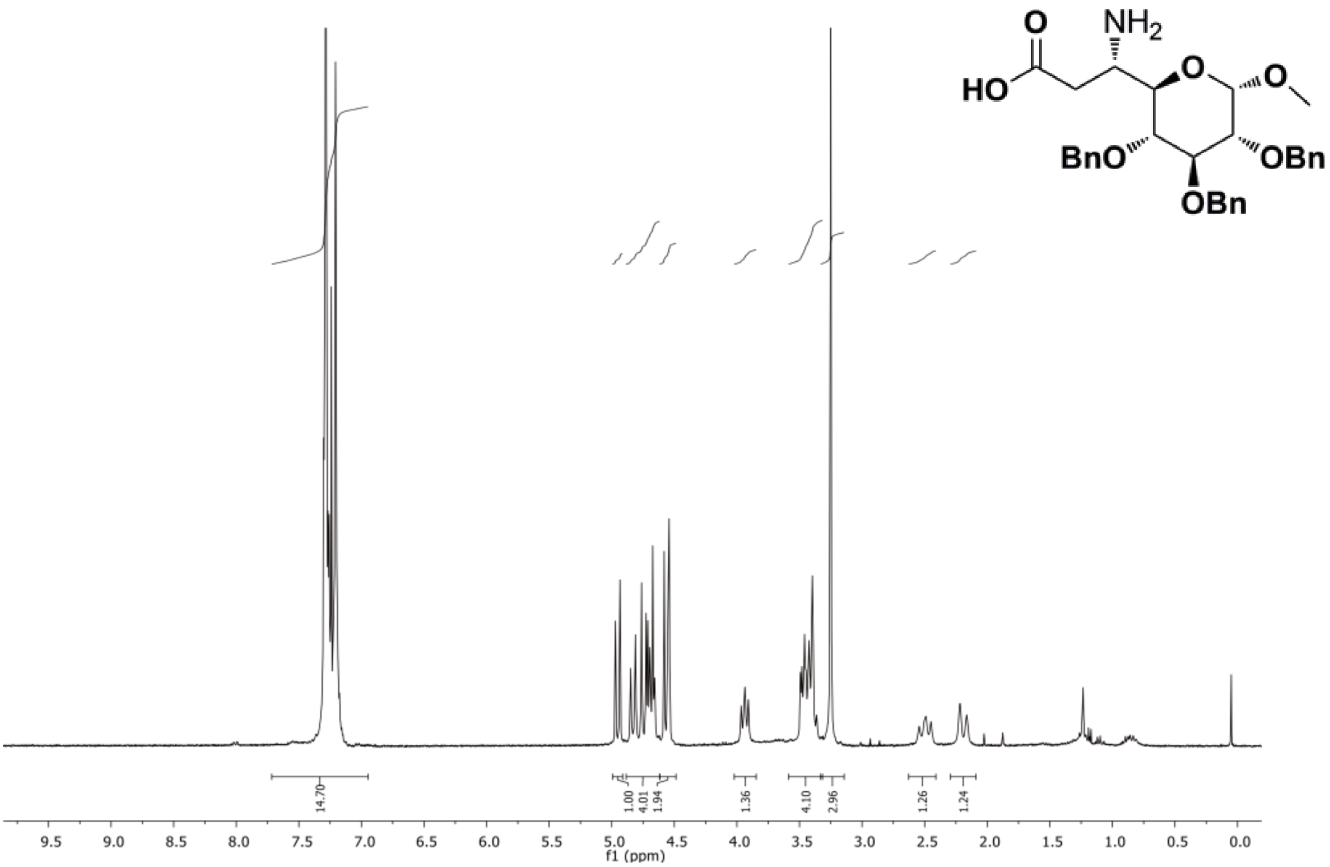


$^{1}$H NMR (300 MHz, $CDCl_3$ + $D_2O$) spectrum of compound **11a**

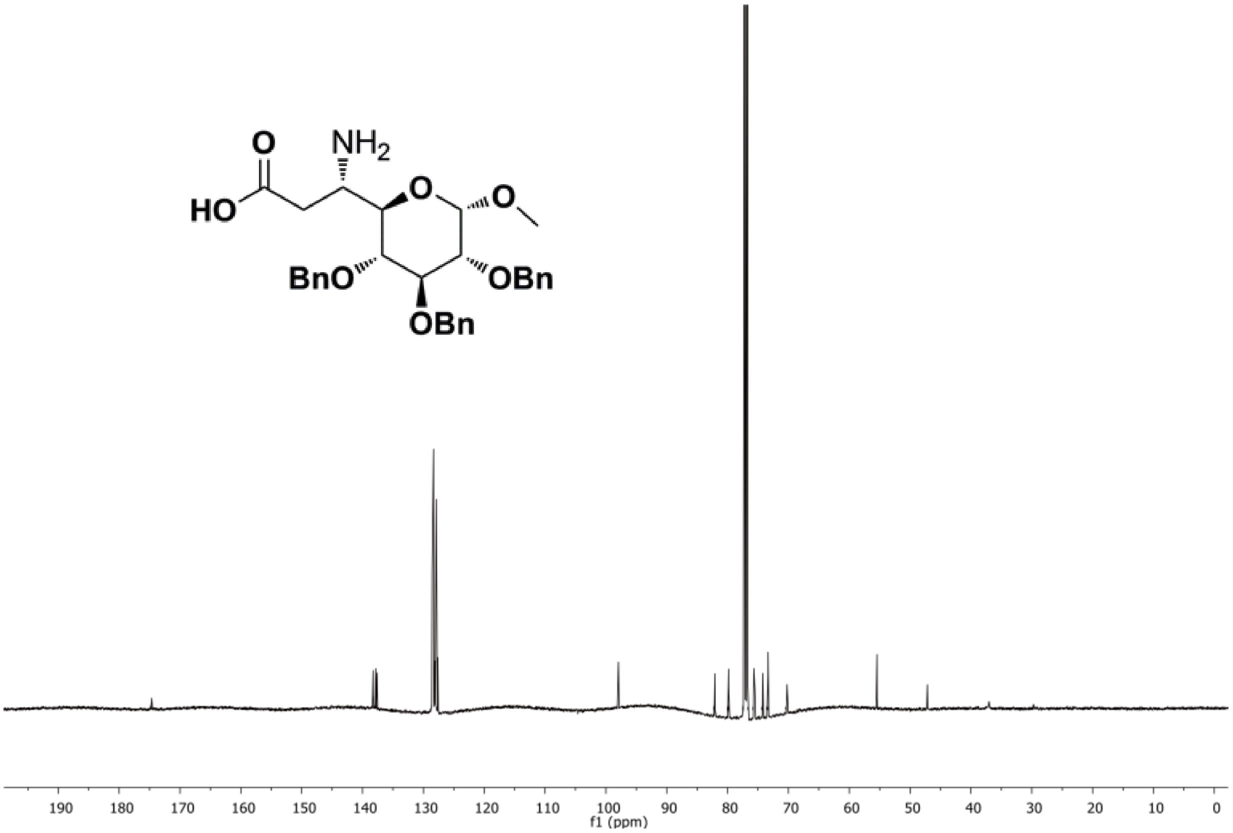


$^{13}$C NMR (125 MHz, $CDCl_3$ + $D_2O$) spectrum of compound **11a**

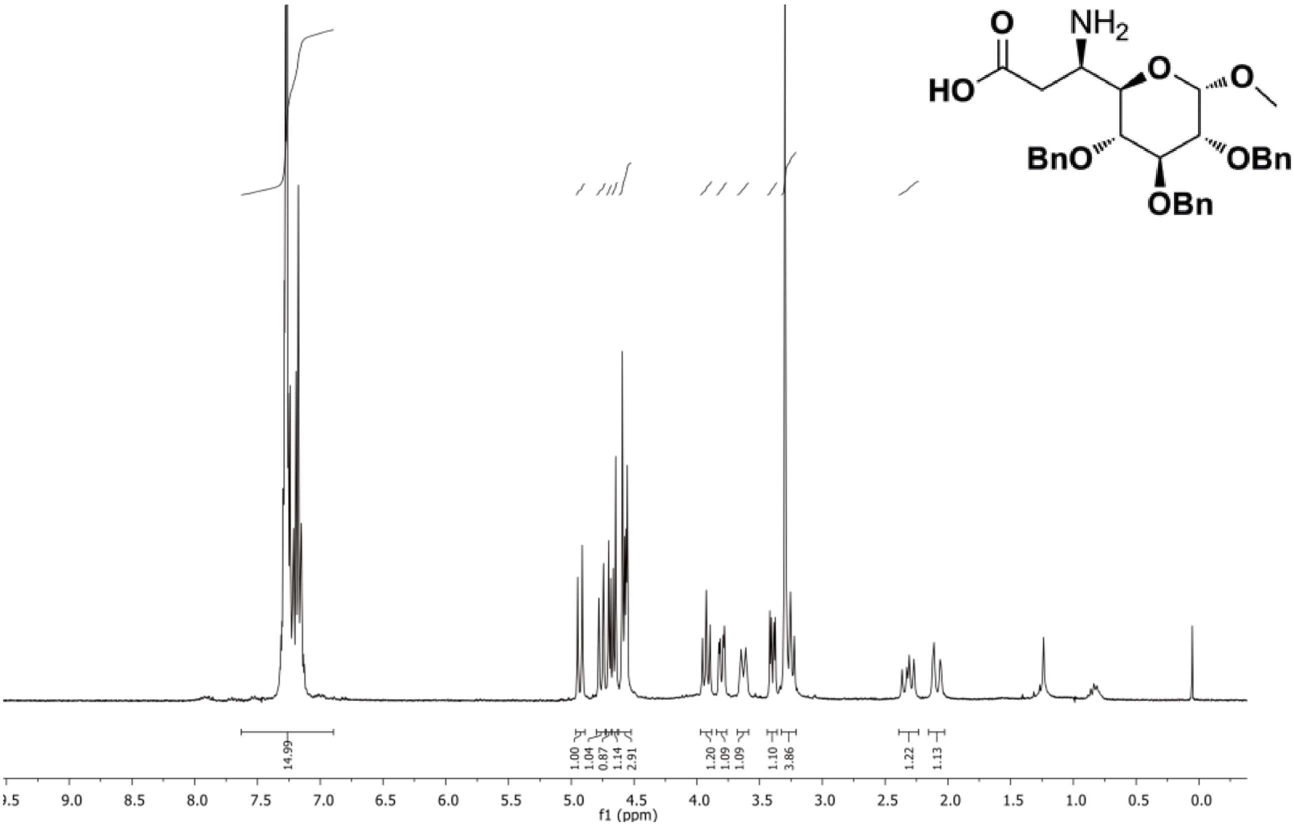


$^{1}$H NMR (300 MHz, $CDCl_3$ + $D_2O$) spectrum of compound **11b**

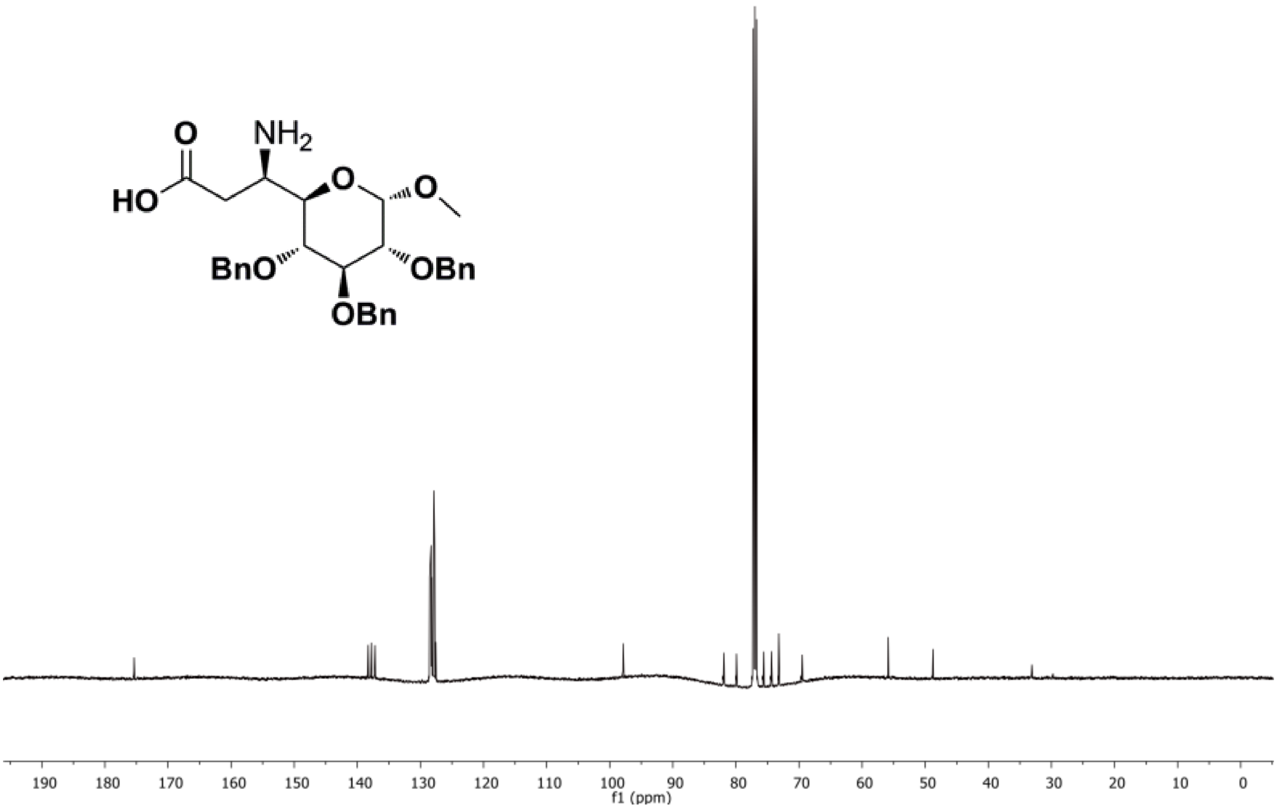


$^{13}$C NMR (125 MHz, $CDCl_3$ + $D_2O$) spectrum of compound **11b**

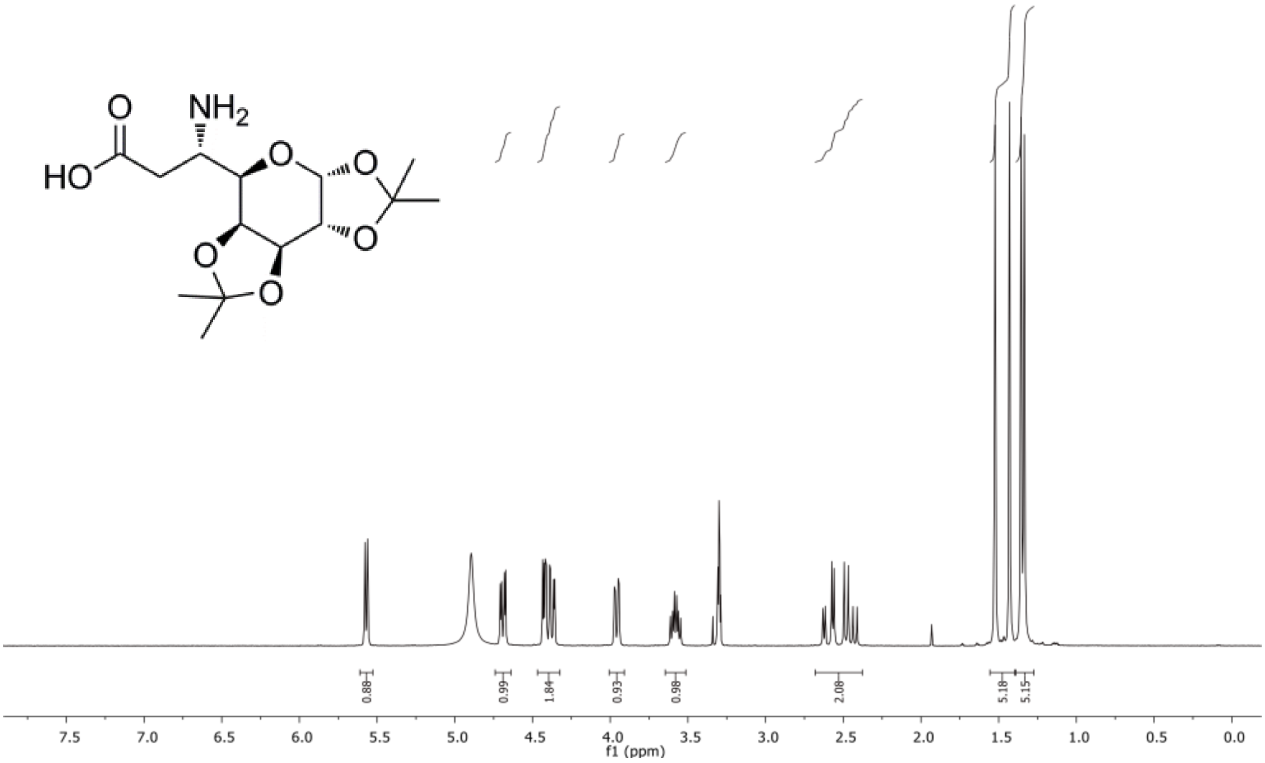


$^{1}H$ NMR (300 MHz, $CD_3OD$) spectrum of compound **11c**

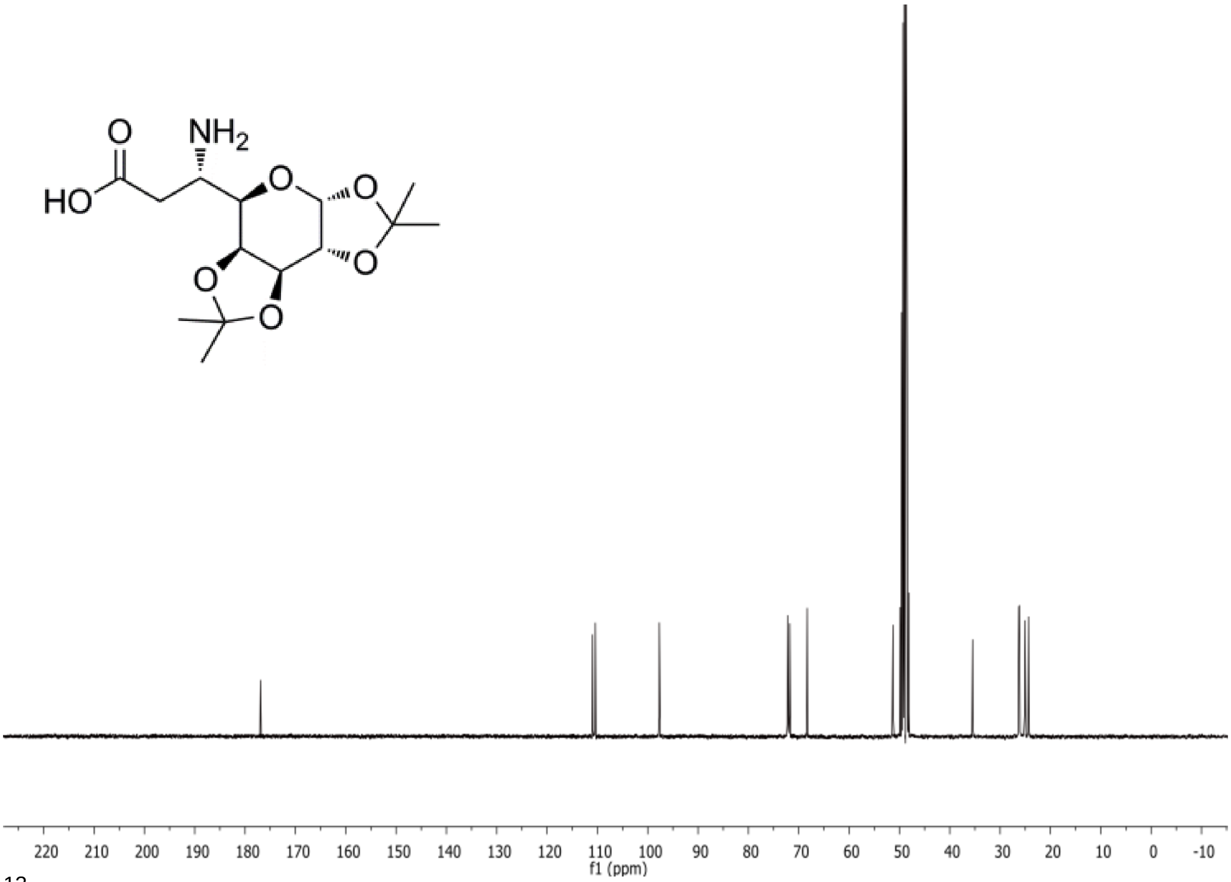


$^{13}C$ NMR (125 MHz, $CD_3OD$) spectrum of compound **11c**

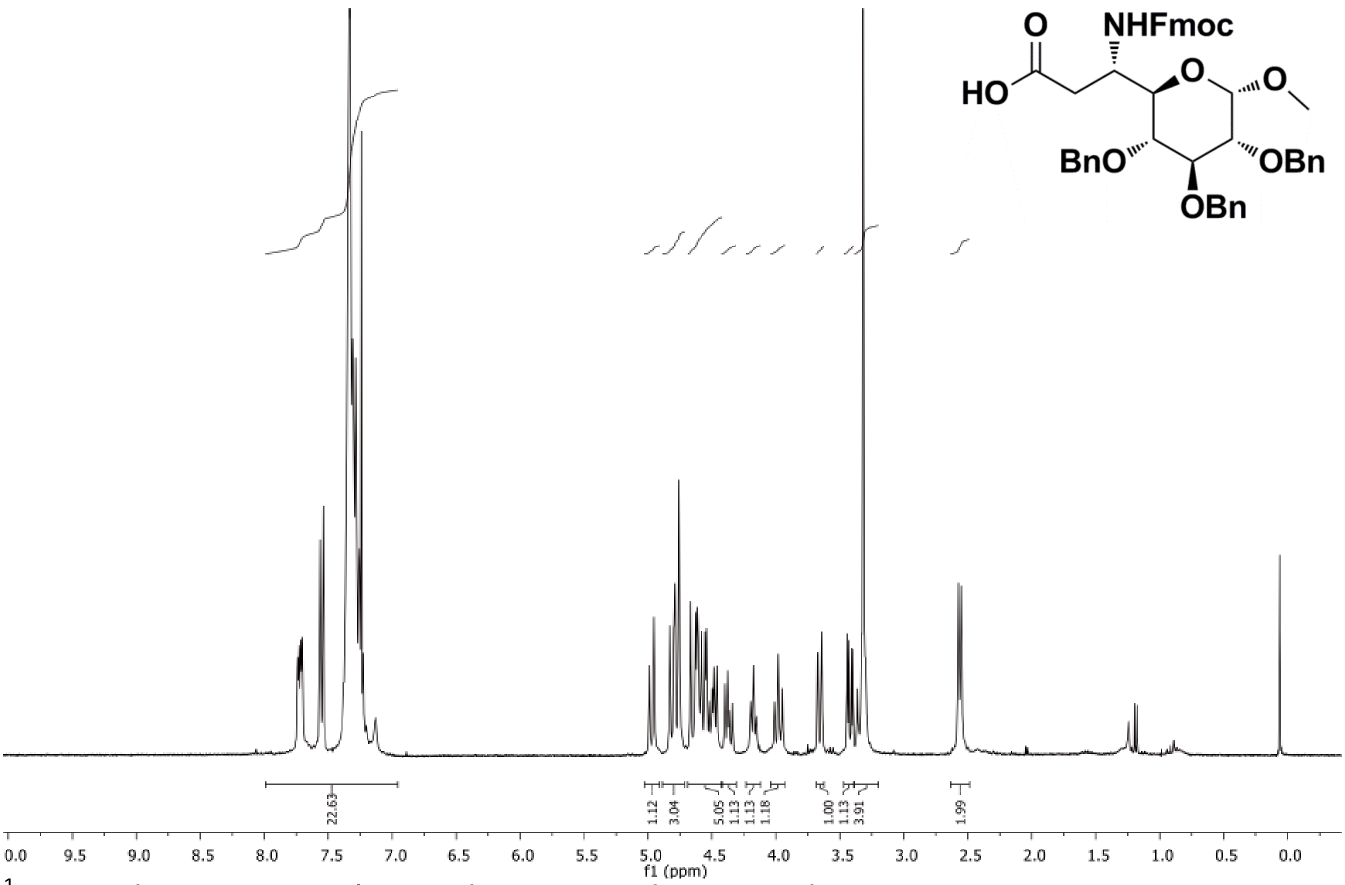


$^{1}H$ NMR (300 MHz, $CDCl_3$ + $D_2O$) spectrum of compound **12a**

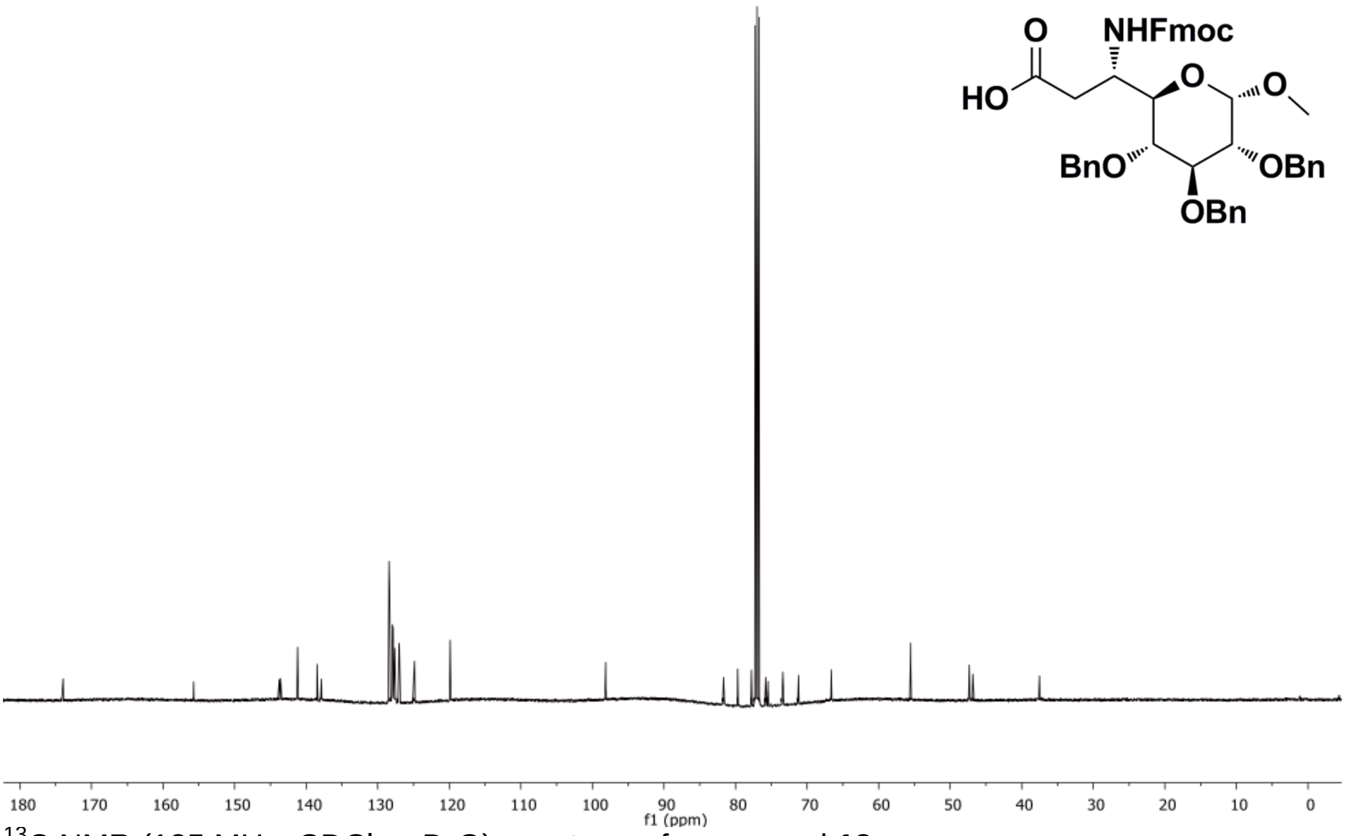


$^{13}C$ NMR (125 MHz, $CDCl_3$ + $D_2O$) spectrum of compound **12a**

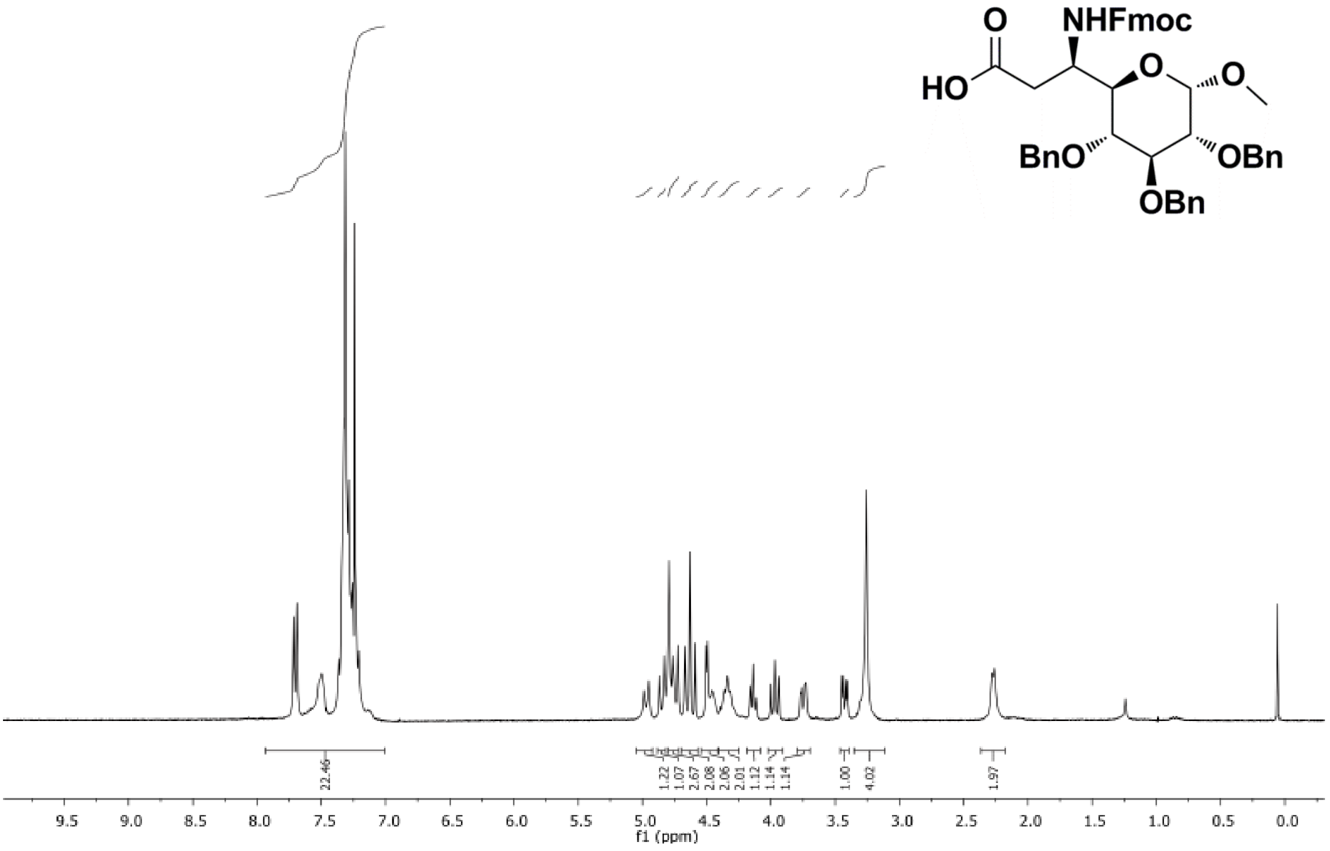


$^{1}$H NMR (300 MHz, $CDCl_3$ + $D_2O$) spectrum of compound **12b**

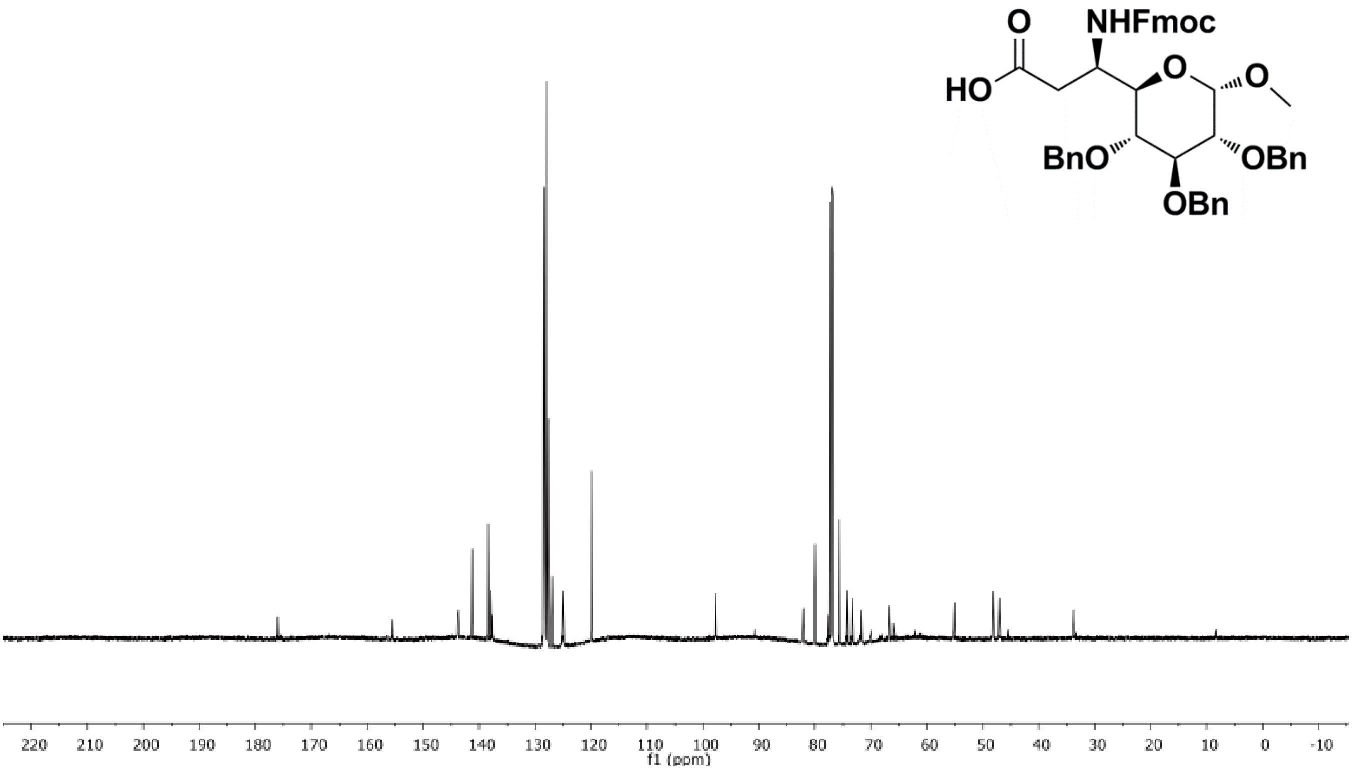


$^{13}$C NMR (125 MHz, $CDCl_3$ + $D_2O$) spectrum of compound **12b**

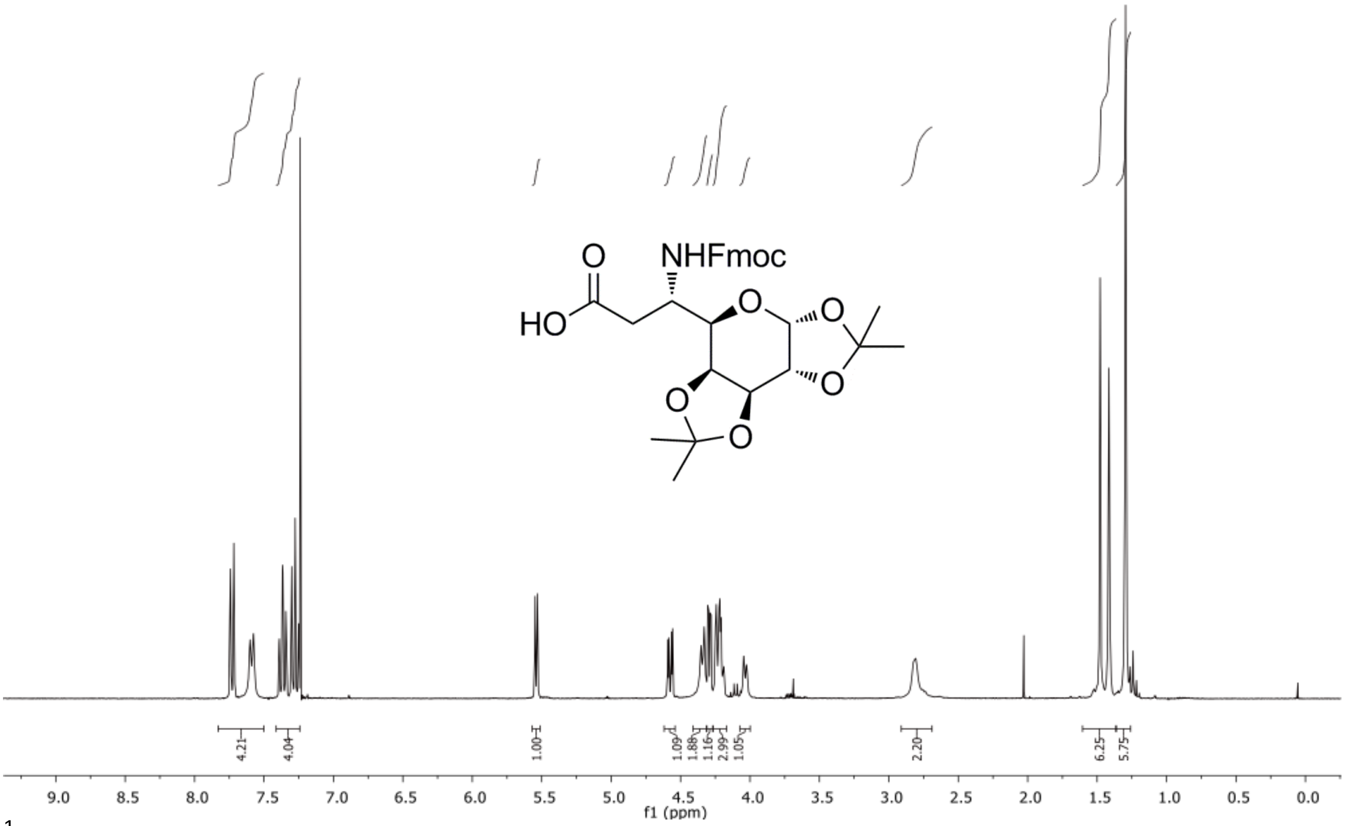


$^{1}$H NMR (300 MHz, $CDCl_3$ + $D_2O$) spectrum of compound **12c**

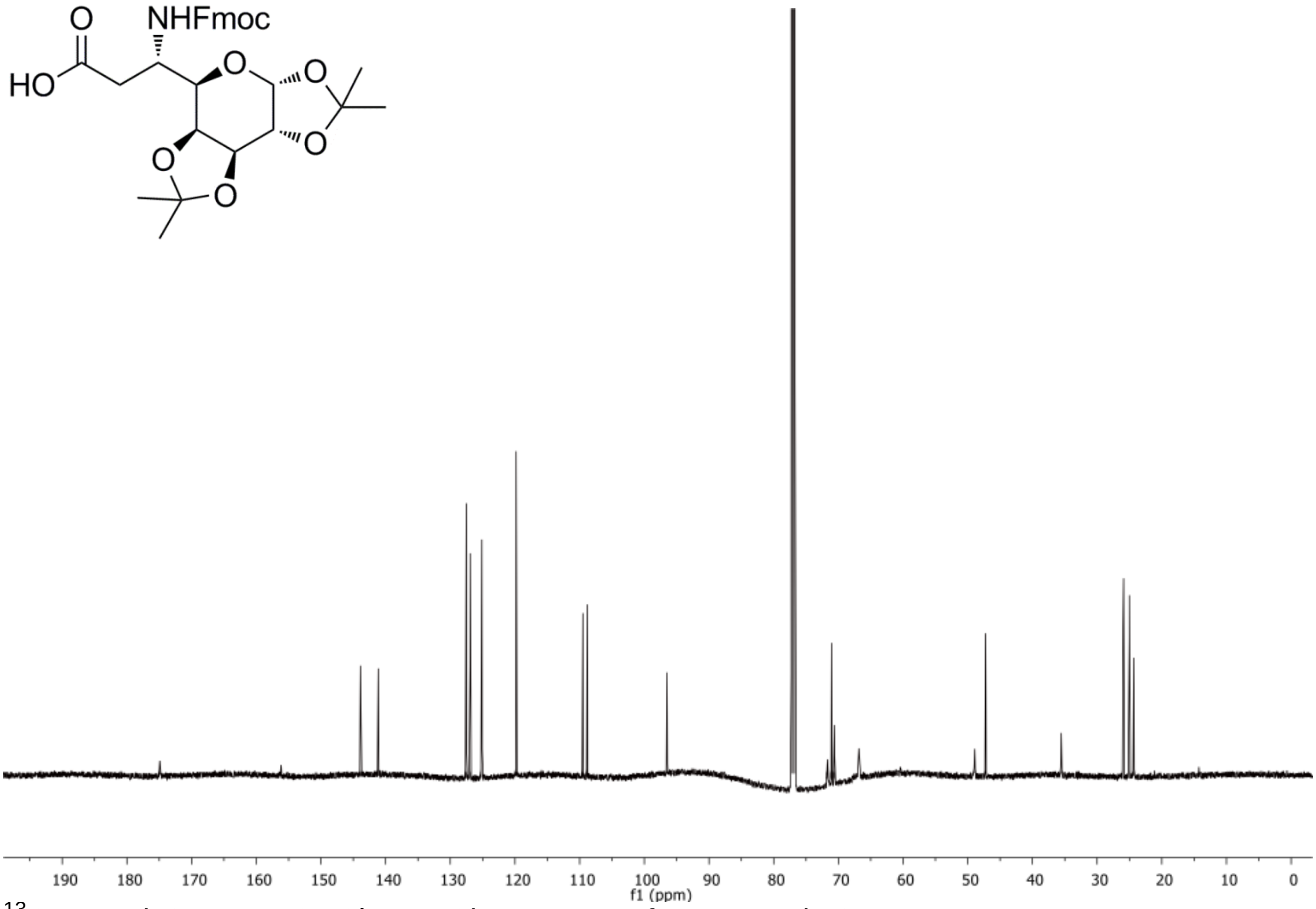


$^{13}$C NMR (125 MHz, $CDCl_3$ + $D_2O$) spectrum of compound **12c**

# 3. HPLC traces of purified β-glycopeptides 1–8

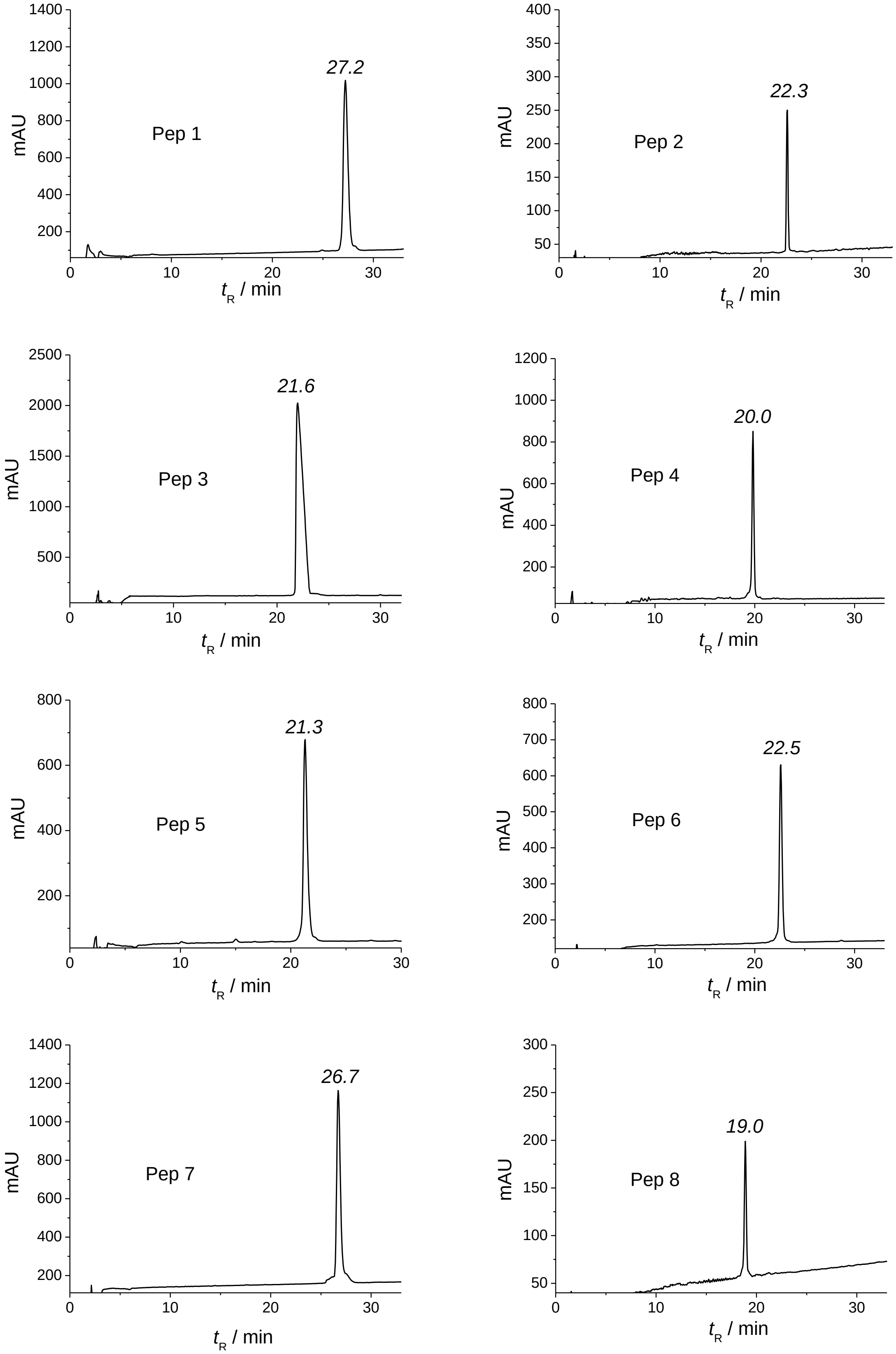

## 4. Crystallographic data of compound 10b

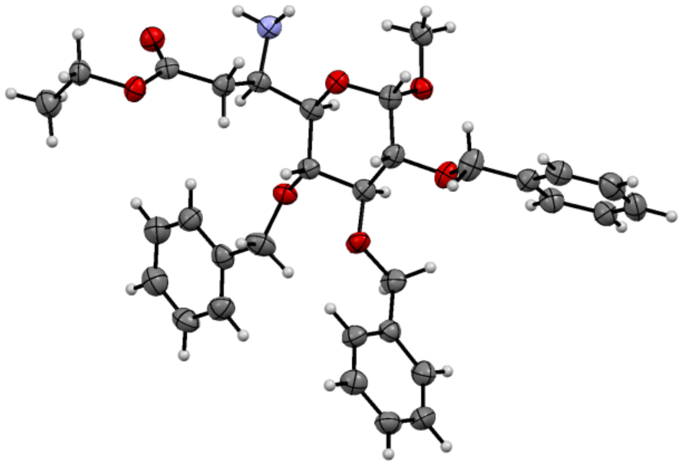

ORTEP diagram of compound **10b.**

**Crystal structure determination:**

The crystal structure of **10b** was determined using single-crystal X-ray crystallography at 100 K. The crystal was desiccated, split and pseudomerohedrally twinned. The twin fraction refined to 0.335. Diffraction data were measured using Cu Kα radiation from a rotating anode source on a SMART 6000 CCD area detector mounted on a Bruker three-circle diffractometer. Integration was done using SAINT [S4], absorption correction and scaling using SADABS [S5], space group determination and data reduction using XPREP [S4]. The structure was solved using direct methods as implemented in SHELXT, with four molecules in the asymmetric unit, two of which are partly disordered. Full-matrix least-squares refinement against $F^2$ was performed using SHELXL-2013 [S6], using RIGU [S7] and SADI restraints for the disordered parts. Model building was done using SHELXLE [S8]. Absolute structure was determined using Parsons´s method [S9], with the Flack parameter refining to a value of 0.001(41).

**Table 1**: Crystal Data and Details of the Structure Determination.
for: 25 P 21 R1 = 0.0432

**Crystal Data:**

| | |
|---|---|
| Formula | C32 H39 N O7 |
| Formula Weight | 549.64 |
| Crystal System | monoclinic |
| Space group | P21 (No. 4) |
| a, b, c [Angstrom] | 9.945(2) 27.644(6) 20.661(4) |
| alpha, beta, gamma [deg] | 90 90.17(3) 90 |
| V [Ang**3] | 5680(2) |
| Z | 8 |
| D(calc) [g/cm**3] | 1.286 |
| Mu(CuKa) [ /mm ] | 0.733 |
| F(000) | 2352 |

**Data Collection:**

| | |
|---|---|
| Temperature (K) | 100 |
| Radiation [Angstrom] | CuKa 1.54178 |
| Theta Min-Max [Deg] | 1.6, 68.4 |
| Dataset | -10: 11 ; -33: 33 ; -23: 24 |
| Tot., Uniq. Data, R(int) | 148369, 19728, 0.052 |
| Observed data [I > 2.0 sigma(I)] | 18576 |

**Refinement:**

| | |
|---|---|
| Nref, Npar | 19728, 1602 |
| R, wR2, S | 0.0432, 0.1198, 1.16 |
| w = S^2^(FO^2^)+(0.0365P)^2^+3.6091P] | WHERE P=(FO^2^+2FC^2^)/3 ' |
| Max. and Av. Shift/Error | 0.00, 0.00 |
| Flack x | 0.001(41) |
| Min. and Max. Resd. Dens. [e/Ang^3] | -0.24, 0.24 |


S1 Schinnerl, M.; Murrary, J. K.; Langenhan, J. M.; Gellman, S. H. *Eur. J. Org. Chem.* **2003,** *4,* 721–726.

S2 Arvidsson, P. I.; Frackenpohl, J.; Seebach, D. *Helv. Chim. Acta.* **2003,** *86,* 1522–1553.

S3 Chakraborty, P.; Diederichsen, U. *Chem.–Eur. J.* **2005,** *11,* 3207–3216

S4 APEX2, SAINT and XPREP, Bruker AXS Inc., Madison, WI, 2009.

S5 G. M. Sheldrick, SADABS, University of Göttingen, Göttingen, Germany, 2012.

S6 G. M. Sheldrick, *Acta Crystallogr*. **2008**, *A 64*, 112–122.

S7 A. Thorn, B. Dittrich, G. M. Sheldrick, *Acta Crystallogr*. **2012**, *A 68*, 448–451.

S8 C. Huebschle, G. M. Sheldrick, B. J. Dittrich, *J. Appl. Crystallogr*. **2011**, *44*, 1281–1284.

S9 S. Parsons, H. D. Flack, T. Wagner, *Acta Cryst.* **2013**, *B69*, 249-259.
